# Modeling and Optimization of a Molecular Biocontroller for the Regulation of Complex Metabolic Pathways

**DOI:** 10.3389/fmolb.2022.801032

**Published:** 2022-03-29

**Authors:** Yadira Boada, Fernando N. Santos-Navarro, Jesús Picó, Alejandro Vignoni

**Affiliations:** Synthetic Biology and Biosystems Control Lab, Institut d’Automàtica i Informàtica Industrial, Universitat Politècnica de València, Valencia, Spain

**Keywords:** metabolic pathway, dynamic regulation, biomolecular antithetic controller, extended biosensor, tuning, gene circuit parts, multiobjective optimization, modeling biological systems

## Abstract

Achieving optimal production in microbial cell factories, robustness against changing intracellular and environmental perturbations requires the dynamic feedback regulation of the pathway of interest. Here, we consider a merging metabolic pathway motif, which appears in a wide range of metabolic engineering applications, including the production of phenylpropanoids among others. We present an approach to use a realistic model that accounts for *in vivo* implementation and then propose a methodology based on multiobjective optimization for the optimal tuning of the gene circuit parts composing the biomolecular controller and biosensor devices for a dynamic regulation strategy. We show how this approach can deal with the trade-offs between the performance of the regulated pathway, robustness to perturbations, and stability of the feedback loop. Using realistic models, our results suggest that the strategies for fine-tuning the trade-offs among performance, robustness, and stability in dynamic pathway regulation are complex. It is not always possible to infer them by simple inspection. This renders the use of the multiobjective optimization methodology valuable and necessary.

## 1 Introduction

Microbial cell factory development using metabolic engineering seeks to obtain high levels of products of interest through genetic modification of microorganisms. Natural cells use complex regulatory networks to preserve robust growth and endure environmental changes by dynamically adapting cell metabolism (Liu et al., 2018). These regulation strategies are the long-term result of evolution. In most cases, they are not compatible with the addition of exogenous genes highly expressed to reach the production levels demanded by the industry. Constraint-based steady-state models of metabolism using only stoichiometric information and some basic information about the enzyme regulation have proved very valuable in providing predictions on maximum theoretical yields, optimal flux distribution to maximize flux towards some metabolite reaction bottlenecks and required ways of intervention on gene expression, leading to fluxes towards final products that achieve specified levels in productivity, titer and yield (Otero-Muras and Carbonell, 2021). This approach seeks the careful optimal selection of the constant expression levels of the exogenous genes in the pathway of interest and the endogenous ones with relevant interactions. Yet, as it is a static regulation approach, it fails to address the problem’s dynamic and highly uncertain nature. Indeed, the static strategy to regulate a metabolic pathway relies on an optimization process that is tailor-made for a particular situation, and therefore it is not able to respond to cell and environmental changes occurring during fermentation in a bioreactor (Wehrs et al., 2019).

Considering the metabolic network dynamics allows better analysis of the sensitivity of the metabolites or fluxes of interest to the optimal enzymatic intervention points under different environmental situations. Dynamic network models, from grey-box to black-box ones, of scales ranging from a subset of pathways to genome-scale, have been used to this end (Otero-Muras and Banga, 2017; Li et al., 2018; Yang et al., 2019; Lo-Thong et al., 2020). The optimal intervention points and intervention strategies (required up- or down-regulation) can be assessed using sensitivity analysis methods like metabolic control analysis (Lo-Thong et al., 2020) dynamic optimization (Otero-Muras and Banga, 2017; Li et al., 2018; Yang et al., 2019) and optimal control principles (Tsiantis and Banga, 2020). Thus, these methods address the fundamental problem of determining the structure of (optimal) control intervention points in complex metabolic networks. Yet, there are no generally applicable algorithms for designing metabolic dynamic feedback regulation systems to date. The regulation topology is generally pathway-specific, depending on both the potential presence of toxic pathway intermediates and the pathway topology (Hartline et al., 2020). Several typical metabolic topology motifs are usually considered: linear, branched, and merging (Blair et al., 2012). Most existing work has dealt with the dynamic regulation of linear pathways (Oyarzún and Stan, 2013; Liu and Zhang, 2018) or branched ones (Liu et al., 2018).

Once the optimal signals to be feedback and the intervention points are obtained, the problem of designing and tuning the proper dynamic feedback regulation biomolecular controller remains. Achieving robust optimal production in microbial cell factories requires considering the dynamic regulation of the pathway of interest. Dynamic feedback regulation constitutes a very interesting strategy to construct pathways with the ability to self-tune upon changing environmental conditions and to overcome many of the ongoing challenges faced in metabolic engineering (Liu and Zhang, 2018; Hartline et al., 2020). For example, it is often challenging to find the proper enzyme levels that maximize production while avoiding pathway bottlenecks or the accumulation of toxic intermediates. Feedback control circuits can solve these problems by dynamically changing enzyme expression in response to metabolic inputs and continuously regulating the activity in the pathway in response to either intracellular or bioreactor perturbations. This enables the industry to attain higher process performance indices than static regulation (Stevens and Carothers, 2015).

Despite the growing number of reported successful cases, engineering dynamic feedback control strategies in biological applications remains a major challenge (Gao et al., 2019). Model-based design, which leverages control engineering principles, can provide a powerful formalism to design dynamic feedback regulation circuits. This, together with the tools of synthetic biology, can lead to robust and efficient microbial production at the industrial level (Liu et al., 2018; Segall-Shapiro et al., 2018).

Here, we consider the design and tuning of a biomolecular controller for the dynamic feedback regulation of a merging metabolic pathway. Since we restrict to a single metabolic pathway, determining the dynamic regulation topology, i.e., the feedback variable and the intervention point, could be made by simple inspection and previous knowledge of the system. In this metabolic motif, two substrates, the primary precursor and an essential metabolite, are converted to an intermediate product which is subsequently transformed into a target product. The secondary essential metabolite plays an additional role in cell metabolism in many practical situations. Therefore, it is subject to environmentally-induced fluctuations. Over-expressing the enzyme that synthesizes this secondary metabolite or redirecting the flux towards it is not feasible in cases where its accumulation is toxic for the cell, leading to growth inhibition. This is the situation encountered in applications like the production of phenylpropanoids of industrial interest, e.g., naringenin (Sheng et al., 2020).

In previous work, we considered the problem of designing a dynamic regulation topology for the production of naringenin while coping with fluctuations in malonyl-CoA, the secondary essential metabolite (Boada et al., 2020). This work considers a detailed model of the whole system, including the metabolic pathway, the extended biosensor, and the molecular biocontroller. We address the problem of optimal choice (tuning) of the biocontroller and the biosensor components in the dynamic regulation topology. In particular, we considered a realistic model for the antithetic controller together with an extended biosensor based on the QdoR Transcription Factor (TF) that accounts for a straightforward *in vivo* implementation of the system. This gives us more information than the simplistic models of the antithetic biocontroller used in the literature that do not consider fundamental aspects like:

• non-linearities in the promoters. In the simplest models, the expression of proteins and sigma factors is always proportional to the number of transcription factors, i.e., there is no saturation of the promoters.
• formation of the antithetic complex, and the unbinding reaction of the complex.
• dilution rate of all the species due to cell growth. It is known that the dilution destroys the perfect adaptation property of the antithetic biocontroller, introducing a steady-state error. As we comment later, this forces us to use more than one objective to optimize.

Multiobjective optimization has already been demonstrated to be an appropriate tool for characterization of gene circuit parts (Boada et al., 2019a; Boada et al., 2019b), and for the design of gene circuits with the desired behavior (Boada et al., 2016; Boada et al., 2017b; Boada et al., 2021). Here, we present an approach to use multiobjective optimization for the optimal tuning of the gene circuit parts composing the biocontroller and biosensor in a dynamic metabolic regulation feedback loop. We show how this approach can deal with the trade-offs between the performance of the regulated pathway and robustness to fluctuations in the secondary metabolite. We also highlight that performance indices must include the standard steady-state industrial ones (e.g., titer) and indices related to the time-response transient (i.e., stability). As the complexity of the dynamic biocontrollers and biosensors integrated into the feedback loop regulation increases, the stability and transient performance issues that high order dynamics introduce must be taken into account. In this work, we consider, on the one hand, the case where having transcription factor (TF) based biosensors of the target product is not always possible. As an alternative, extended TF-based biosensors can be used, where an additional pathway is introduced from the target product to be regulated to a measurable metabolite (Boada et al., 2020). Yet, these extended biosensors present extra dynamics in the feedback loop. On the other hand, to regulate the amount of enzyme that catalyzes the conversion from the two precursor substrates into the product naringenin, we consider the use of the antithetic controller, a biomolecular integral feedback controller that achieves quasi-perfect adaptation (Briat et al., 2016; Aoki et al., 2019).

We first show our approach using a simple illustrative pathway that captures the essential topological features of merging metabolic pathways. We use a feedback regulation strategy encompassing a simple TF-based biosensor to obtain readouts of the product and a simplified model of the biomolecular antithetic controller. In this case, the final titer of the target product and the robustness to fluctuations in the secondary metabolite are evaluated. Then, we consider a detailed model of the metabolic merging pathway of naringenin, the biocontroller, and the extended biosensor of naringenin production that we previously introduced in (Boada et al., 2020). In this case, we use a more realistic model of the antithetic biocontroller. The extra dynamics introduced by both the extended biosensor and the biocontroller force us to consider the transient dynamics of the regulated feedback loop in the design process. A library of designs is obtained, each one corresponding to a different trade-off.

## 2 Results

### 2.1 Tuning the Dynamic Regulation of a Merging Metabolic Pathway

**FIGURE 1:**
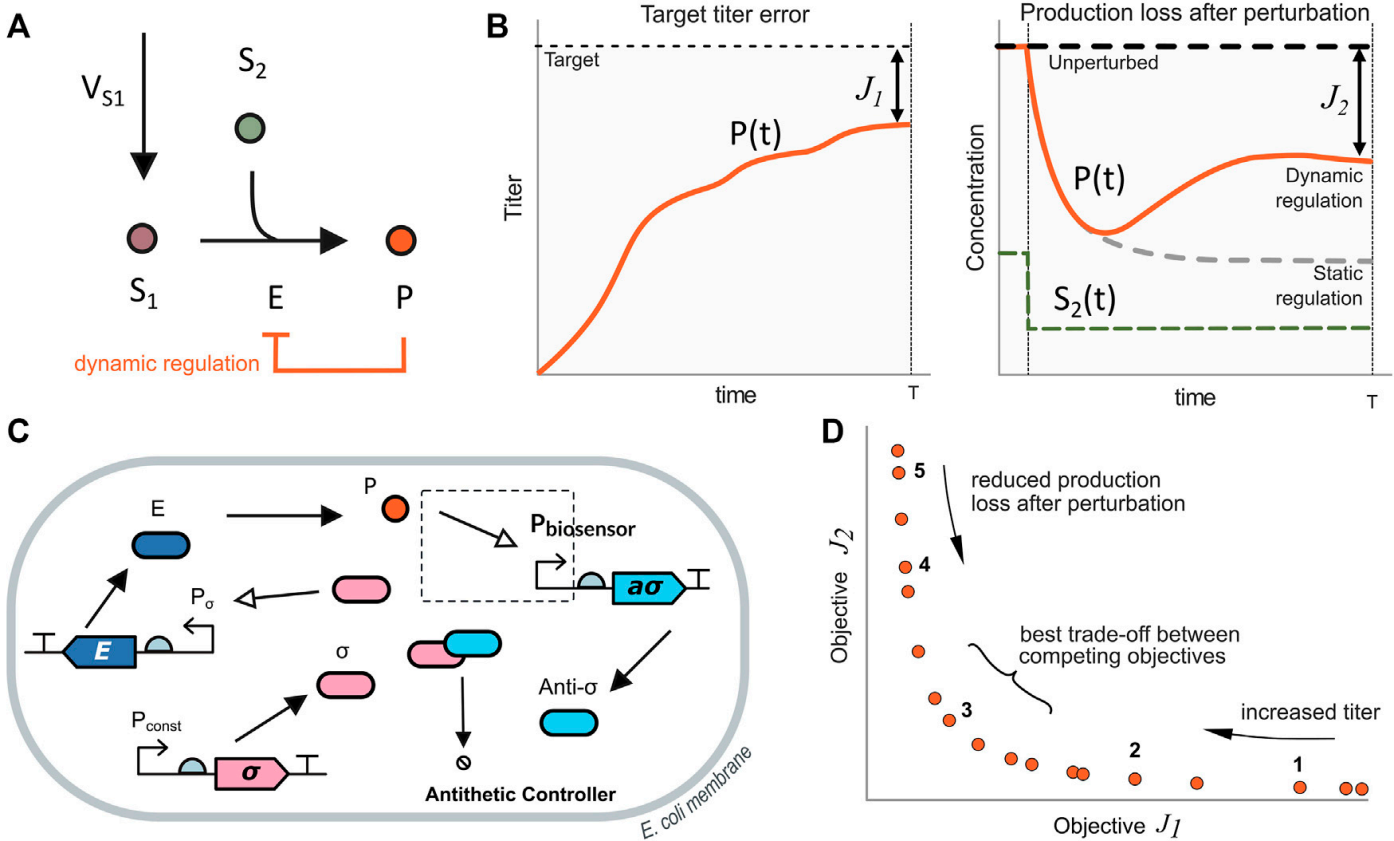
Illustrative model system. **(A)** Metabolic pathway for the production of metabolite *P*. The main substrate *S*_1_ and the secondary one *S*_2_ are converted into the product *P* by the catalyst enzyme *E*. In the static regulation strategy (black lines), the expression level of the enzyme *E* remains constant in time. Conversely, in the dynamic regulation strategy (orange line), the expression of the enzyme *E* depends on the amount of product *P*. **(B)** Objective functions employed in this work for the maximization of the production up to a target value (*J*_1_) together with the minimization of the production loss after perturbations (*J*_2_) as defined in Eq. 9, 10 **(C)** Biosensor and antithetic controller configuration for dynamic pathway regulation. The amount of free *σ* molecule determines the expression of the enzyme *E*. A TF-based biosensor detects the product levels and counteracts expressing the anti-*σ* molecule. When the amount of *P* decreases, the controller reduces the amount of expressed anti-*σ*, thus increasing the amount of free *σ* to up-regulate the enzyme *E*. **(D)** Pareto front of optimal solutions for the dynamical pathway regulation case. Solutions on the right side have large titer target error *J*_1_ (i.e., lower titer) and a small production loss after perturbation *J*_2_ (i.e., higher titer after the perturbation). Moving along the Pareto front towards the left, the titer target error decreases, and the production loss increases. Solutions in the middle of the Pareto front have the best trade-off between the competing objectives *J*_1_ and *J*_2_.

To illustrate our approach’s broad scope and usefulness, we first study a basic metabolic pathway that contains the main common features of a typical merging motif. As shown in Figure 1A (black lines), we consider the production of a product metabolite *P* from a precursor substrate *S*_1_ and a secondary substrate *S*_2_. The reaction is catalyzed by the enzyme *E*. This metabolic pathway can be described using the following model:

$$\frac{dS_{1}}{dt}=V_{S_{1}}-V_{S_{1},S_{2}}-\mu S_{1}$$ (1)

$$\frac{dP}{dt}=V_{S_{1},S_{2}}-\mu P$$ (2)

$$\frac{dX}{dt}=\mu X\left(1-\frac{X}{X_{\text{max}}}\right)$$ (3)

Where *S*_*i*_ and *P* are the amount of substrates and product. *X* is the number of cells in the population. *S*_1_ is the primary substrate, and *S*_2_ is the secondary substrate. The first order dilution term represents the effect of cell growth on the amount of substrates and products, being *μ* the specific growth rate. X_max_ accounts for the maximum growing capacity of the population. The metabolic fluxes are given by the kinetic terms:

$$V_{S_{1}}=K_{S_{1}}$$ (4)

$$V_{S_{1},S_{2}}=k_{\text{cat}}E\frac{S_{1}S_{2}}{K_{{\text{mS}}_{1}}\;K_{{\text{mS}}_{2}}+K_{{\text{mS}}_{2}}S_{1}+K_{{\text{mS}}_{1}}S_{2}+S_{1}S_{2}}$$ (5)

Where we assume that the uptake of the precursor *S*_1_ has constant rate $K_{S_{1}}$ (4), and the substrate *S*_2_ is normally available at non-limiting amount. The flux $V_{S_{1},S_{2}}$ is described by means of the Michaelis-Menten kinetics in Eq. 5, where *E* is the amount of enzyme catalyzing the pathway, k_cat_ is the enzyme catalytic rate and $K_{{\text{mS}}_{i}}$ are the Michaelis-Menten constants for the substrates.

In the case of static pathway regulation (Figure 1A, black line), the flux $V_{S_{1},S_{2}}$ has a constant maximum value determined by the amount of the constitutively expressed heterologous enzyme *E*. As its expression level is independent of any metabolite in the pathway, the production of *P* is affected in the presence of a sudden change in the availability of the secondary substrate *S*_2_, as shown in the right plot of Figure 1B in dashed grey lines.

On the contrary, in the case of dynamic pathway regulation (Figure 1A in orange line), the level of expression of the enzyme *E* depends on the amount of the product metabolite. A biosensor provides product metabolite readouts, and a biomolecular controller changes the enzyme expression level as a function of the difference between the current amount of product and the target one encoded in the controller. Thus, when there is a change in the secondary substrate, the production of the metabolite *P* is affected but can recover (up to some extent) closer to its previous value (Figure 1B, right plot, solid orange line).

Different control architectures can be implemented with combinations of activation and repression feedback loops. Here, we focus on a class of biomolecular controller, the antithetic controller (Aoki et al., 2019), that allows for quasi-perfect adaptation.

To gain an initial understanding of the design trade-offs in the dynamic control of the merging metabolic pathway motif, we first consider a simplified version of the antithetic controller regulating the amount of enzyme *E* using a simple TF-based biosensor to obtain readouts of *P* (Figure 1C).

The control action is encoded in the amount of free *σ* molecules that activate the expression of the enzyme *E* through its promoter *P*_*σ*_. We modeled the promoter using a generalized Hill function as in (Boada et al., 2020), including the effect of the plasmid copy number on the promoter activation function. The resulting dynamics of the amount of enzyme *E* is:

$$\frac{dE}{dt}=C_{\text{N}}a_{0}+C_{\text{N}}a_{1}\frac{\sigma^{2}}{k_{\text{d}20}C_{\text{N}}^{2}+\sigma^{2}}-\left(d_{\text{E}}+\mu \right)E$$ (6)

The acting molecule *σ* is constitutively expressed (thus encoding for sort of a target set-point value) and binds the anti-*σ* molecule to form an inactive complex, effectively reducing the amount of free *σ*. The resulting dynamics of the amount of free *σ* molecules is:

$$\frac{d\sigma }{dt}=C_{\text{N}}k_{\sigma }-\gamma \sigma a\sigma -\left(d_{\sigma }+\mu \right)\sigma$$ (7)

Next, a TF-based biosensor detects the product *P* expressing the anti-*σ* molecule as a function of the product amount. A constitutively expressed Transcription Factor (TF) (equation omitted for brevity) binds to the product *P* inducing the expression of the anti-*σ* molecule. The dynamics of the amount of anti-*σ* molecules is:

$$\frac{da\sigma }{dt}=C_{{\text{N}}_{\text{a}\sigma }}k_{\text{a}\sigma }\frac{C_{\text{N}}^{2}{\left(1+\frac{P}{k_{\text{dp}}}\right)}^{2}}{C_{\text{N}}^{2}{\left(1+\frac{P}{k_{\text{dp}}}\right)}^{2}+{\left(TF\right)}^{2}}-\gamma \sigma a\sigma -\left(d_{\text{a}\sigma }+\mu \right)a\sigma$$ (8)

When the amount of *P* decreases, so does the amount of expressed anti-*σ*, thus increasing the amount of free *σ* molecules and, this way, up-regulating the expression of the enzyme *E*. Next, we consider the optimal tuning of the gene circuit parts composing the antithetic biocontroller and the TF-based biosensor.

To characterize the trade-offs between reaching the desired titer target for *P* together with reducing the production loss after a perturbation on the level of the secondary metabolite *S*_2_, as illustrated in Figure 1B, we considered two objective functions. For the first one, we looked for the difference between the titer of the product *P* in the bioreactor and the desired target value (*J*_1_). For the second one, we focus on the production loss (amount of product expressed per cell) after a perturbation on *S*_2_ (*J*_2_). The corresponding expressions for both objectives to be jointly minimized are:

$$J_{1}=\left|Target-KP_{\text{unperturbed}}\left(T\right)\right|,\;\;\;\left(\text{target titer error}\right)$$ (9)

$$J_{2}=\left|\frac{P_{\text{unperturbed}}\left(T\right)-P_{\text{perturbed}}\left(T\right)}{P_{\text{unperturbed}}\left(T\right)}\right|,\;\;\;\left(\text{production loss after perturbation}\right),$$ (10)

Where *P*_unperturbed_(*T*) is the product amount at the end of the experiment (time T), *K* is a conversion constant from the amount of product to titer, *P*_perturbed_(*T*) is the amount of product after a perturbation in the secondary metabolite. As *J*_1_ describes the difference between the desired target titer and the actual one, lower values of *J*_1_ correspond to larger titers. On the other hand, *J*_2_ is related to the loss in production after a perturbation. Therefore, small values of *J*_2_ correspond to low production loss after a perturbation. That is a better rejection of the perturbation on the secondary metabolite.

Next, we selected the biosensor and biocontroller set of parameters to be tuned. We took into account to what extent these parameters can be changed in the biological implementation of the system at the lab. Thus, we considered the set: expression strength for the enzyme *E*, a_1_; the dissociation constant between *σ* and the enzyme promoter, k_d20_; and the expression strength for anti-*σ*, k_a*σ*_. Additionally, the specific growth rate, *μ*, was included as a decision parameter to account for the dependency of the results on cell growth.

The goal is to obtain a library of possible designs, each one corresponding to a different trade-off between the cost indices *J*_1_, *J*_2_. The resulting solutions are all equally optimal in the sense of Pareto (Boada et al., 2019b). When one of the objectives improves, the others necessarily deteriorate, so selecting the most appropriate solution depends on the designer.

We computed the values of the selected parameters as the solution of the multiobjective optimization problem min (*J*_1_, *J*_2_) subject to biologically plausible bounds on the values of the parameters (see Supplementary Table S1 for a list of the solutions). Thus, for example, we set a growth rate corresponding to doubling times between 25 and 90 min, an upper bound for the dissociation constant of the promoter k_d20_ < 3.5 *μ*M and an upper bound on the maximum enzyme level of 180 *μ*M. The optimization problem was solved using a multiobjective optimization genetic algorithm based on differential evolution. The detailed statement of the optimization problem is described in the Methods section, and the parameters used are in Supplementary Table S2 (Supplementary Material).

**FIGURE 2:**
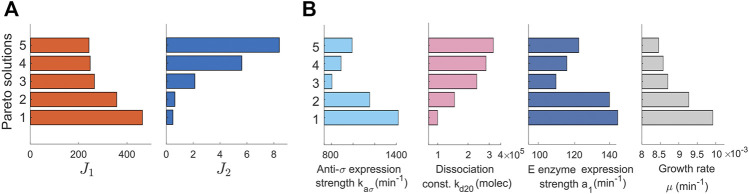
Pareto solutions. Pareto front and Pareto set of selected solutions. **(A)** Pareto front showing the solutions of the multiobjetive problem. The values of the objectives *J*_1_ and *J*_2_ (*x*-axis) are represented for different solutions (*y*-axis). **(B)** The Pareto set represented with a plot for each tuned parameter. The tuned values of the parameters (*x*-axis) are shown for each selected Pareto solution (*y*-axis). The set of solutions constitutes the library of biocontrollers and biosensors obtained with the multiobjective optimization tuning process.

The resulting Pareto front, Figure 1D, has three distinct regimes: 1) large titer target error and low production loss, 2) small titer target error and high production loss, and 3) the best trade-off regime between the two competing objectives. The convexity of the Pareto front indicates that the optimization problem is well-posed, in the sense that both objective functions oppose each other across the whole space of optimal solutions. We selected five solutions that represent the mentioned regimes. These solutions are highlighted in the Pareto front in Figure 1D. The achieved objective values of the selected solutions are shown in Figure 2A and the corresponding tuned optimal values for the controller and biosensor parameters and the growth rate are shown in Figure 2B (see Supplementary Figure S1, for details on the temporal responses of the selected solutions). Thus, the set of solutions of the optimization constitutes a library of optimally tuned controller-biosensor pairs.

A detailed inspection of the library of controller and biosensor pairs obtained (Figure 2B) reveals that the relations between parameters and objectives are not necessarily monotonous (Supplementary Table S1, Supplementary Material). For example, the dissociation constant k_d20_ must be chosen smaller to reduce the production loss after perturbation (*J*_2_). Yet, there is no monotonous trend in neither the anti-*σ* expression strength *k*_*aσ*_ nor in the *E* enzyme expression strength *a*_1_.

Altogether these results suggest that strategies for fine-tuning the trade-off between target titer error and production loss in dynamic pathway regulation are complex and impossible to obtain by simple inspection even for a simplified case, rendering the use of the multiobjective optimization methodology not only helpful but necessary.

### 2.2 Model of the Dynamic Regulation of the Naringenin Metabolic Pathway

Naringenin is a flavonoid compound predominantly found in grapefruits and oranges. It has been reported to have many pharmacological properties, including anti-dyslipidaemic, anti-obesity and anti-diabetic (Liu et al., 2008; Zygmunt et al., 2010; Rahigude et al., 2012). Flavonoids are an essential subclass of phenylpropanoids, an important family of plant natural products with diverse uses as food supplements, antioxidants, flavoring and flavoring agents, pharmaceuticals, insecticides and colorants. Significant market opportunities clearly exist for flavonoids with enhanced bioavailability and bioactivity profiles that are used, among others, as flavorings and bioactive compounds for nutraceutical applications.

**FIGURE 3:**
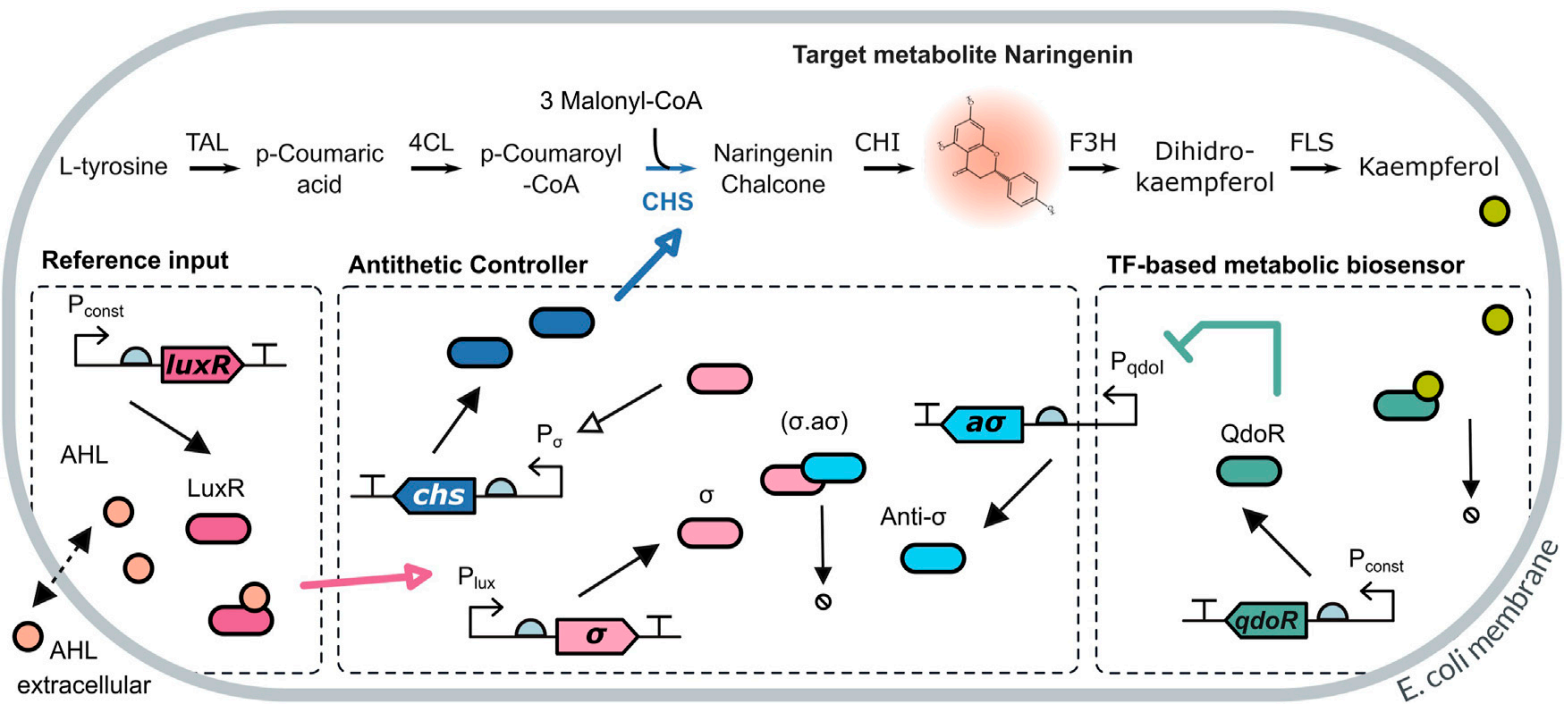
Dynamic pathway regulation scheme for the naringenin pathway. The target metabolite naringenin is produced from L-tyrosine in four enzymatic steps, including a merging step catalysed by the enzyme naringenin chalcone synthase (CHS) which incorporates the secondary metabolite malonyl-CoA. The production level of naringenin is readout using a metabolic extended TF-based biosensor through the downstream metabolite kaempferol. This is sensed using the QdoR TF-based biosensor and feeds back to an antithetic biomolecular controller. The controller can be activated upstream by means of the external inducer AHL. Its actuating signal overdrives the basal constitutive expression of the CHS enzyme in the pathway in order to compensate for malonyl-CoA depletion.

The naringenin pathway has four enzymatic steps from the *L*-tyrosine precursor (see Figure 3). The third step, catalysed by the naringenin chalcone synthase enzyme (CHS) requires the co-substrate malonyl-CoA, an essential metabolite that is used in fatty acid production and plays an important role in cell metabolism. Intracellular concentrations of malonyl-CoA are typically low (4–40 *μ*M in *E. coli*) (Xu et al., 2014; Johnson et al., 2017). Moreover, its concentration is subject to fluctuations caused by cell environmental heterogeneity. Accumulation of malonyl-CoA is toxic for the cell, so that over-expressing it is not a feasible solution.

We considered a detailed model of the naringenin pathway by obtaining the mass balance equations of the enzyme-catalyzed reactions of the metabolic pathway from *L*-tyrosine to naringenin (see Figure 3). From the mass balance equations, we obtained the set of rate Eq. 11. The rate equations include the dilution effect of cell replication at a specific growth rate *μ*.

$$\begin{array}{ll} \frac{dLt}{dt} & =V_{0}-V_{Lt}-\mu Lt\\ \frac{dpC}{dt} & =V_{Lt}-V_{pC}-\mu pC\\ \frac{dpA}{dt} & =V_{pC}-V_{pA,Ma}-\mu pA\\ \frac{dNc}{dt} & =V_{pA,Ma}-V_{Nc}-\mu Nc\\ \frac{dN}{dt} & =V_{Nc}-V_{N}-\mu N \end{array}$$ (11)

For each reaction, the corresponding flux is *V*_*j*_ (molecules min^−1^). *Lt* is the number of molecules of *L*-tyrosine, *pC* is *p*-coumaric acid, *pA* is *p*-coumaroyl-CoA, *Nc* is naringenin chalcone, and *N* is the target metabolite naringenin. Next, we assumed that the fluxes *V*_*j*_ follow the Michaelis-Menten kinetics (Michaelis and Menten, 1913), and the flux *V*_0_ from the L-tyrosine precursor is kept as a constant, obtaining the equations:

$$\begin{array}{ll} V_{0} & =K_{Lt}\\ V_{Lt} & =k_{\text{catTAL}}TAL\frac{Lt}{K_{\text{mLt}}+Lt}\\ V_{pC} & =k_{\text{cat}4\text{CL}}4CL\frac{pC}{K_{\text{mpC}}+pC}\\ V_{pA} & =k_{\text{catCHS}}CHS\frac{pAMa}{K_{\text{mpA}}\;K_{\text{mMa}}+K_{\text{mMa}}pA+K_{\text{mpA}}Ma+pAMa}\\ V_{Nc} & =k_{\text{catCHI}}CHI\frac{Nc}{K_{\text{mNc}}+Nc}\\ V_{N} & =k_{\text{catF}3\text{H}}F3H\frac{N}{K_{\text{mN}}+N} \end{array}$$ (12)

Where *Ma* is the number of malonyl-CoA molecules naturally available inside the cell, k_catj_ is the catalytic rate of each enzyme (min^−1^), and K_*mj*_ is the Michaelis-Menten constant for each substrate. The enzyme kinetic parameters, detailed in Supplementary Table S4 (Supplementary Material) were obtained from Brenda (Schomburg et al., 2017) and optimized according to the requirements for the pathway implementation in the lab.

Malonyl-CoA is one of the major building blocks for cell metabolism. Its intracellular concentration is tightly regulated and maintained at small amounts (Yang et al., 2015). Therefore, our system, the exogenous naringenin pathway, will compete for this resource. Thus, from the point of view of our system, any variation in the *Ma* level caused by changes in the cell will act as a perturbation signal. We considered a basal value of *Ma* in the mid-range of values reported in the literature (Takamura and Nomura, 1988; Xu et al., 2014; Wu et al., 2015), and avoiding the accumulation of large amounts of intermediate metabolites that may lead to growth inhibition.

The amounts of the enzymes TAL, 4CL, CHI, CHS, and F3H involved in the naringenin pathway were previously optimized so that the flux of precursor L-tyrosine can yield the targeted 1 g/L of naringenin (see Supplementary Table S4, Supplementary Material). Compared to other models, we are explicitly modeling the amount of the enzymes of interest (CHS) as variables of our model to capture the interaction between the genetic control level and the metabolic pathway level.

#### 2.2.1 Feedback Regulation *via* a Metabolic Biosensor and Biocontroller for the Naringenin Pathway

For the naringenin pathway, we implemented a dynamic regulation strategy including an extended biosensor to obtain the readout of naringenin and a biomolecular controller. On the one hand, the TF-based biosensor provides readouts of the amount of naringenin *via* a short metabolic pathway from naringenin to kaempferol (see Figure 3). Kaempferol is the effector flavonoid measured by the biosensor promoter region *P*_*qdoI*_ and the QdoR transcription factor (TF) (Siedler et al., 2014). When kaempferol captures QdoR, the TF is inactivated, and repression of the *P*_*qdoI*_ promoter becomes weaker while leading to an increase of anti-*σ* factor production. In contrast, lower concentrations of kaempferol allow higher amounts of QdoR transcription factor, which inhibits anti-*σ* expression.

For every *i*—cell in the population, the kinetics of the enzyme-catalyzed reactions involved in this extended pathway were modeled using the set of rate Eq. 13:

$$\begin{array}{ll} \frac{d\;Di}{dt} & =V_{N}-V_{Di}-\mu Di\\ \frac{dKa}{dt} & =V_{Di}-\mu Ka\\ \frac{d\;Q}{dt} & =\frac{p_{\text{Q}}C_{\text{N}}k_{\text{Q}}}{{dm}_{\text{Q}}+\mu }-\left(d_{\text{Q}}+\mu \right)Q \end{array}$$ (13)

Where *Di* is the number of molecules of Dihydrokaempferol, *Ka* is kaempferol, the flux *V*_*Di*_ obeys the Michaelis-Menten kinetics $V_{Di}=k_{\text{catFLS}}FLS\frac{Di}{K_{\text{mDi}}+Di}$ , and *Q* is the constitutively expressed QdoR protein. All the parameters are listed in Supplementary Tables S4, S5 (Supplementary Material) and optimized according to the lab implementation and characterization in (Boada et al., 2019b; Dunstan et al., 2020).

The antithetic controller used for the dynamic regulation of naringenin production is depicted in Figure 3. The antithetic motif relies on the annihilation mechanism between both *σ* and anti − *σ* factor proteins. The *σ* factor activity is controlled by the anti − *σ* factor that binds to and keeps the *σ* factor sequestered. The anti − *σ* is only released and de-repressed in response to the QdoR transcription factor. The dimer formed by the LuxR protein and AHL lactone activates the *P*_*LuX*_ promoter, inducing the synthesis of *σ* factor. The externally added concentration of AHL acts as the desired reference input for naringenin production. We do not assume the AHL concentration needed to set the desired value for naringenin must be equal to this one—implying an unnecessary metabolic burden—but simply proportional. Free *σ* factor binds to the *P*_20_ promoter to activate expression of the naringenin chalcone synthase CHS, which subsequently converts p-Coumaroyl-CoA and malonyl-CoA into the naringenin precursor. In other words, the CHS enzyme represents the controller output signal.

Considering the same assumptions as those to derive the TF-based biosensor model, the dynamics of the antithetic controller for every cell is given by the following set of equations:

$$\begin{array}{ll} \frac{d\sigma \cdot a\sigma }{dt} & =\frac{k_{-c}}{k_{\text{dc}}}\sigma \;a\sigma -k_{-c}\sigma \cdot a\sigma -\left(d_{\text{c}}+\mu \right)\sigma \cdot a\sigma \\ \frac{d\sigma }{dt} & =\frac{p_{\sigma }C_{\text{N}}k_{\sigma }}{{dm}_{\sigma }+\mu }\left(\alpha +\frac{\left(1-\alpha \right)A^{2}}{k_{\text{dlux}}{\left(\frac{k_{\text{d}2}C_{\text{N}}}{R}\right)}^{2}+A^{2}}\right)-\frac{k_{-c}}{k_{\text{dc}}}\sigma \;a\sigma +k_{-c}\sigma \cdot a\sigma -\left(d_{\sigma }+\mu \right)\sigma \\ \frac{da\sigma }{dt} & =\frac{p_{\text{a}\sigma }C_{{\text{N}}_{\text{a}\sigma }}k_{\text{a}\sigma }}{{dm}_{\text{a}\sigma }+\mu }\left(\alpha +\frac{\left(1-\alpha \right){\left(k_{\text{dq}}C_{\text{N}}\right)}^{2}{\left(k_{\text{dk}}+\mathrm{Ka}\right)}^{2}}{{\left(k_{\text{dq}}C_{\text{N}}\right)}^{2}{\left(k_{\text{dk}}+Ka\right)}^{2}+{\left(k_{\text{dk}}Q\right)}^{2}}\right)-\frac{k_{-c}}{k_{\text{dc}}}\sigma a\sigma \\ & +k_{-c}\sigma \cdot a\sigma -\left(d_{\text{a}\sigma }+\mu \right)a\sigma \\ \frac{dCHS}{dt} & =\beta \frac{p_{{\text{H}}_{\text{c}}}C_{\text{Nh}}k_{\text{H}}}{{dm}_{\text{H}}+\mu }+\frac{p_{\text{H}}C_{\text{Nh}}k_{\text{H}}}{{dm}_{\text{H}}+\mu }\left(\alpha +\frac{\left(1-\alpha \right)\sigma^{2}}{k_{\text{d}20}{\left(k_{\text{d}\sigma }C_{\text{Nh}}\right)}^{2}+\sigma^{2}}\right)-\left(d_{\text{H}}+\mu \right)CHS \end{array}$$ (14)

Where *σ* ⋅ *aσ* is the amount of molecules from the generated complex after *σ* sequestration, *σ* and *aσ* are the factor and its cofactor, respectively. All the parameters are listed in Supplementary Table S5 (Supplementary Material).

As in (Boada et al., 2020), the desired naringenin set-point is regulated by the external addition of AHL and the constitutive expression of the LuxR protein. The passive diffusion of extracellular AHL inside the cell was modeled as a reversible pseudo-reaction using mass-action kinetics (Boada et al., 2017a). This resulted in the set of Eq. 15:

$$\begin{array}{ll} \frac{dR}{dt} & =\frac{p_{\text{R}}C_{\text{N}}k_{\text{R}}}{{dm}_{\text{R}}+\mu }-\left(d_{\text{R}}+\mu \right)R\\ \frac{dA}{dt} & =D\left(V_{\text{c}}A_{e}-A\right)-\left(d_{\text{A}}+\mu \right)A\\ \frac{dA_{e}}{dt} & =D\left(-xV_{\text{c}}A_{e}+\overset{x}{\underset{i=1}{\sum }}A\right)-d_{\text{Ae}}A_{e}\\ \frac{dx}{dt} & =\mu \left(1-\frac{x}{x_{\text{max}}}\right)x \end{array}$$ (15)

Where *R* is the number of molecules of LuxR, *A* and *A*_*e*_ are the intra and extracellular AHL molecules, respectively, the term $V_{\text{c}}=\frac{V_{\text{cell}}}{V_{\text{ext}}}$ is the ratio between the cellular and the culture volumes; and *x* is the number of cells. The parameters are also listed in Supplementary Table S5 (Supplementary Material).

**FIGURE 4:**
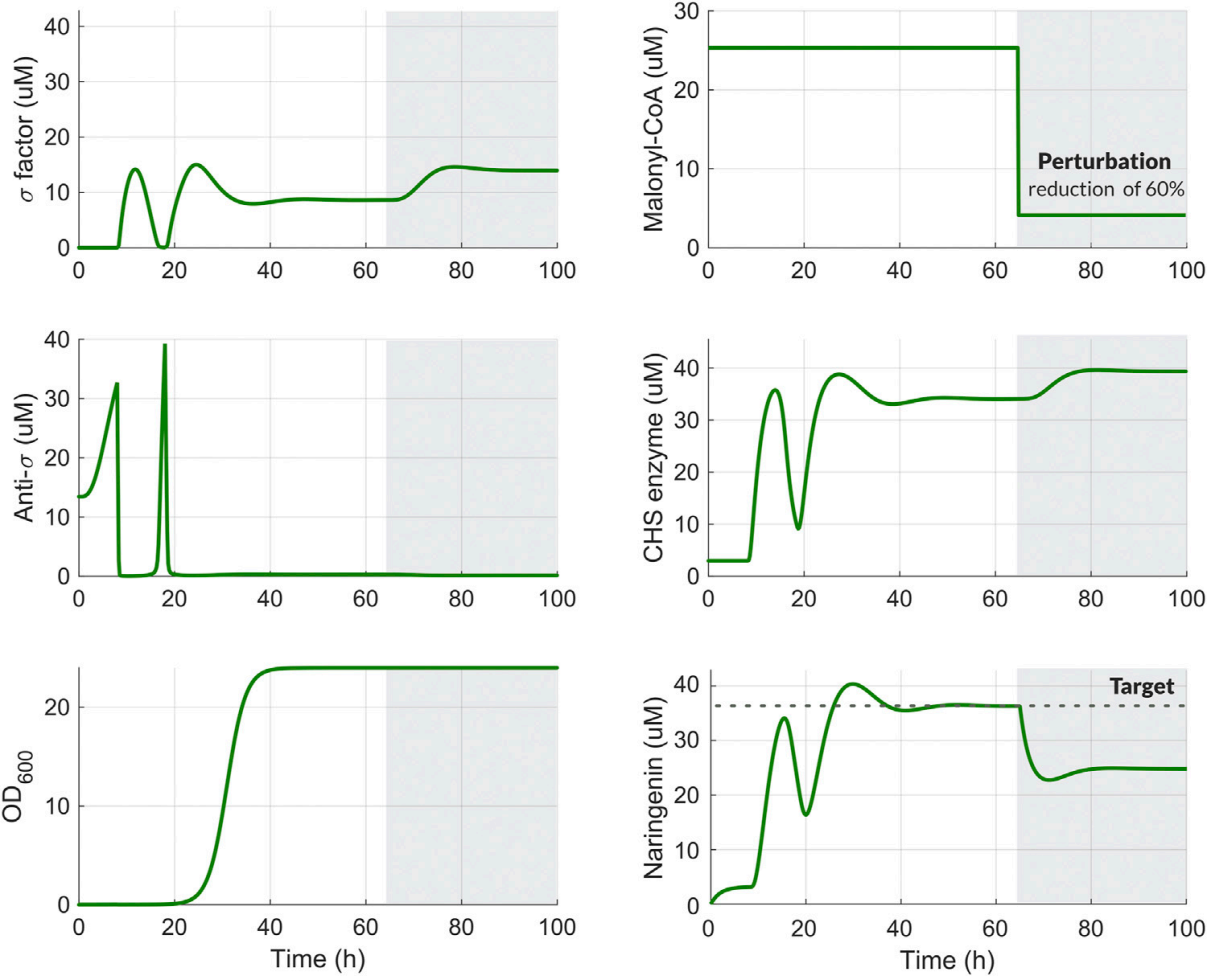
Temporal response of the system including the dynamic pathway regulation scheme for the naringenin pathway. Time-course variation in the biocontroller species (*σ* and Anti‐*σ*), malonyl-CoA secondary substrate, CHS enzyme, naringenin, and cellular growth (OD) before (white background) and after 60% reduction in malonyl-CoA availability (grey background). After the perturbation, the amount of naringenin begins to decline until it recovers steadily thank to the transient increase in the amount of the CHS enzyme. This increase is generated by the activation of factor when the perturbation occurs.

Using the set of preliminary parameters in Supplementary Table S5, we ran computational simulations to obtain the temporal response of the system to perturbation in Malonyl-CoA. Once the production of the target metabolite naringenin reached steady-state, we introduced a perturbation in the availability of Malonyl-CoA at 65 h of 60%. After that, the amount of *σ* factor increases leading to an increased expression of the enzyme CHS. This results in a slight increase in the naringenin production. However, as seen in Figure 4, there is room for improvement. This will be the goal obtained in the next section by means of the optimal tuning of the regulation strategy.

### 2.3 Optimal Tuning of the Dynamic Regulation for Naringenin Production

Having developed a detailed and realistic model of the pathway dynamic regulation, we optimally tuned the controller and biosensor components of the dynamically regulated metabolic pathway that produces naringenin in *Escherichia coli* (*E. coli*).

We first established a baseline production pathway for naringenin, with the basal level of the CHS enzyme provided by a constitutive promoter. On top of this, the feedback control loop regulates the total level of expression of CHS to give a robust response to the fluctuations in the secondary co-substrate malonyl-CoA availability and drive the production of naringenin up to the target industrially relevant value of 1 g L^−1^.

Next, we used our multiobjective optimization approach to find a library of optimal biocontroller and biosensor pairs for the dynamic regulation of the naringenin pathway. As in the previous example, we aim to determine controller and biosensor designs that allow reaching a target titer of naringenin production while minimizing the production loss after perturbations in the secondary metabolite. However, the extra dynamics introduced by the extended biosensor used in this case and the ones introduced by using a more realistic model of the biomolecular antithetic controller must be considered. These extra dynamics force us to consider the transient behavior of the regulated feedback loop in the design process to evaluate the overall stability properties of the designed system. Thus, we defined the following three objective functions to be minimized:

$$J_{1}=\left|Target-KN_{\text{unp}}\left(T\right)\right|,\;\;\;\left(\text{target titer error}\right)$$ (16)

$$J_{2}=\left|\frac{N_{\text{unp}}\left(T\right)-N_{\text{pert}}\left(T\right)}{N_{\text{unp}}\left(T\right)}\right|,\;\left(\text{production loss after perturbation}\right),$$ (17)

$$J_{3}=\left(\text{Number of oscillations in }\sigma \right),$$ (18)

Where N_*unp*_ and N_*pert*_ are the amount of naringenin before and after the malonyl perturbation at the time T, respectively, and *J*_3_ is an indirect measure of the frequency and damping factor of the transient in the antithetic biocontroller (see Methods for a detailed description).

A preliminary parameter sensitivity analysis of the biological parts from the biocontroller and the biosensor revealed six parameters to be tuned in the optimization process (Boada et al., 2020): the translation rates of both anti-*σ* and the CHS enzyme, p_a*σ*_ and p_H_; the plasmid copy numbers $C_{{\text{N}}_{\text{a}\sigma }}$ and $C_{{\text{N}}_{\text{h}}}$ ; the *σ* anti-*σ* complex dissociation rate, k_−c_; and the dissociation constant between *σ* and the CHS enzyme promoter k_d20_. The majority of these parameters are also easy to tune in the real implementation in the lab. Additionally, as in the simplified example before, we also considered the growth rate, *μ*, as a decision parameter. We computed them as the optimal solutions of the multiobjective problem min (*J*_1_, *J*_2_, *J*_3_). The details of the optimization problem can be found in the Methods section and details on the obtained solutions can be found in the Supplementary Material, including the list of the solutions in Supplementary Table S3, the representation of the Pareto front in Supplementary Figure S2, the Pareto set in Supplementary Figure S3 and the temporal responses in Supplementary Figure S4.

**FIGURE 5:**
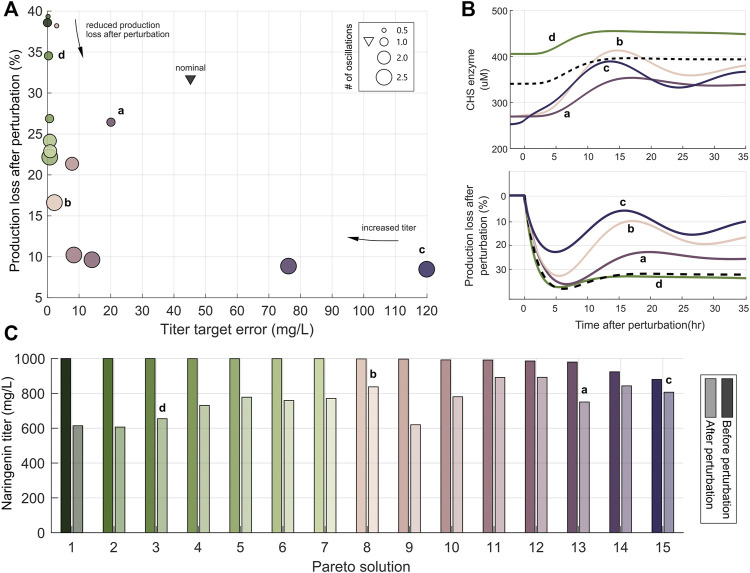
Optimal controller and biosensor tuning in the naringenin dynamic pathway regulation. **(A)** Pareto front of the optimal solutions. The *x*-axis is the objective *J*_1_ (titer target error), the *y*-axis is the objective *J*_2_ (production loss after perturbation), and the size of the circles represent the objective *J*_3_ (number of oscillations) that take into account the transient response of the controller to a perturbation. Solutions along the Pareto front are identified with color ranging from dark green to dark violet as the values of objective *J*_1_ increase. Green solutions have smaller target error than pink/purple/violet ones. The black triangle represents the preliminary not optimized configuration. Solutions *a*-*d* are representative of the different zones along the Pareto front. **(B)** Time response of the selected solutions. Top plot: response of the CHS enzyme after a perturbation in the secondary metabolite Malonyl-CoA. Bottom plot: time response of the production loss after perturbation with respect to the level achieved before the perturbation for the four selected solutions. In both plots the black dashed line corresponds to the preliminary not optimized solution. **(C)** Naringenin titer before and after perturbation of each one of the Pareto solutions. The color codes are common to all the plots in the figure.

The Pareto front resulting from the optimization is shown in Figure 5A. Several interesting aspects arise from it. First, all the solutions found are better than the preliminary configuration that was not optimized. They have either smaller titer target error or lower production loss after perturbation or both. Second, the relevance of considering the objective *J*_3_, related to the transient characteristics, can be clearly seen. This objective is represented by the size of the circles that correspond to each of the solutions in the Pareto front. Notice that reducing the production loss can be achieved at the cost of increasing the titer error as also seen before in the previous example. But in this case, an increased capacity to reject perturbations also increases the number of oscillations in the response, and thus corresponds to a less marginally stable configuration of the controller.

However, unlike in the previous example, in which the oscillations were no taken into account in the optimization process, now it is possible to obtain a compromise design (see solution *a* in Figure 5) that has small titer error and low number of oscillations (a lightly under-damped response in Figure 5B) at a fairly low production loss cost. Notice that the apparent best trade-off without considering *J*_3_ (with solution *b* as representative) has too an under-damped and lengthy transient, which could be unacceptable in some cases. Extreme solutions like (*c*) which has the lowest production loss or (*d*) with the smallest titer error may be of interest in particular cases when one objective has more practical importance than the others.

**FIGURE 6:**
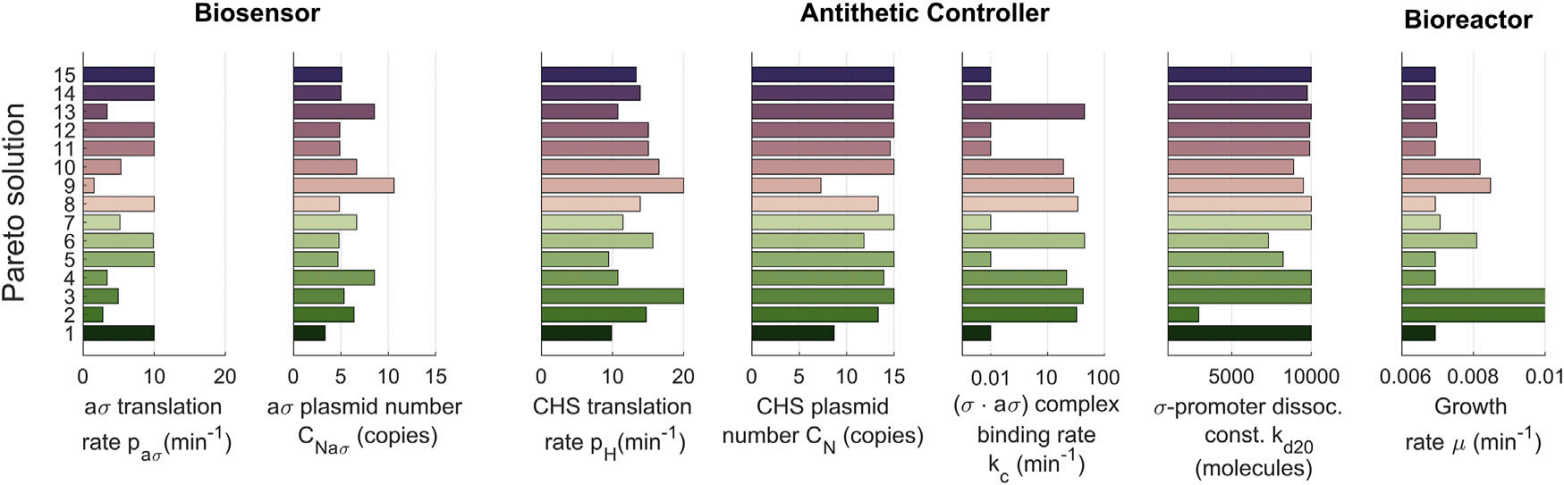
Library of optimal controller-biosensor devices for the dynamic regulation of the naringenin pathway. Pareto set representing the optimal tuned values of the controller-biosensor parameters (decision variables). On the *x*-axis we show the optimal values of the parameters for each solution. Each one of the solutions (in the *y*-axis) constitutes an element of the controller-biosensor library. The color code is the same as the one used in Figure 5.

The trade-off is evident in the case of solutions *b*,*c* and *d* when only looking at the first two objectives. In this situation, solution *b* is the obvious best trade-off between titer error and production loss as it also clearly seen in Figure 5C. However, taking into account for the transient response of the biocontroller (*J*_3_) shown in Figure 5B, solution *a* arises as a better compromise with less under-damped response. A detailed inspection of the library of controller and biosensor pairs obtained (Figure 6) again reveals the complex non monotonous relationship between parameters and objectives.

Taking a deeper look into the obtained library, we can also make an interesting observation: different combinations of parameters result in similar performances. This is nothing more than another evidence of the inherent robustness obtained with negative feedback control. For example, devices 5, 6, and 7 from the library (Figure 6, see Supplementary Figures S2–S4 in the Supplementary Material for more details) have an approximately equivalent performance regarding the three objectives. Still, they significantly differ in their parameter values. Device 6 works in a faster-growing culture without losing titer, with a higher CHS translation rate than the other two devices, but needs a higher biding rate for the (*σ* ⋅a*σ*) complex. Depending on the available biological parts, one implementation can be more feasible than another, increasing the importance of having such a variety of elements in the library.

## 3 Discussion

Dynamic regulation of metabolic pathways is a crucial strategy to achieve optimal production in microbial cell factories while coping with cell and environmental fluctuations. The appropriate dynamic regulation topology will be particular to the topology and characteristics of the metabolic pathway to be regulated. Yet, on the one hand, some basic metabolic motifs that often appear in practical industrial applications and their appropriate dynamic regulation topology can be identified. On the other hand, all dynamic regulation schemes share a set of common features that determine to a great extent the appropriate methodological tools required for the optimal selection of the gene circuit parts composing them. In particular, there is the common need to address a set of multiple goals related to the system’s performance in terms of both the production of the targeted product and the rejection of perturbations affecting it. In addition, the stability issues that arise as a result of using feedback regulation strategies must be addressed. This is even more important as we use complex biomolecular controllers and metabolic extended biosensors that introduce extra dynamics that may compromise the regulated system’s transient time response and stability.

In this work, we have shown the application of a general approach based on multiobjective model-based optimization for building libraries of gene circuit parts that achieve optimal performance of a dynamically regulated merging metabolic pathway. This metabolic motif appears in many situations of practical interest and, in particular, is a pervasive motif in producing phenylpropanoid-derived natural products.

The multiobjective optimization approach obtains devices within resulting libraries with different combinations of parameter values but similar performances. This is another sign of the inherent robustness obtained with negative feedback control. Interestingly, depending on the available biological parts, one implementation can be more feasible than another, increasing the importance of having such a variety of elements in the library.

We used detailed models of the metabolic kinetics and the biosensor and biocontroller dynamics constituting a sort of *in vivo* construction guidelines, as some of the model parameters can be directly related to biological parts or devices in the laboratory. Our results show that using this type of model with enough granularity also forces us to consider the transient and stability issues that are often disregarded.

The need for enough detailed models arguably includes the use of host-aware models. Indeed, the library of designs we obtained might suffer some modifications in case we considered the interactions between the regulated metabolic pathway and the host cell caused by competition for cell resources (Santos-Navarro et al., 2021). Our goal in this work was to present the general multiobjective optimization approach, emphasizing the tuning of the biomolecular controller and biosensor. In any case, the use of host-aware models will not change the general framework; it will only change the obtained solutions.

Altogether our results suggest that strategies for fine-tuning the trade-offs between target performance, robustness, and stability in complex dynamic pathway regulation topologies are intricate and not possible to obtain by simple inspection, rendering the use of the multiobjective optimization methodology not only helpful but necessary. As a consequence, it will not be generally possible to obtain widely applicable optimal simple rules for the design. Instead, the expected outcome of the tuning process should be libraries of gene circuit components that achieve specific trade-offs and specific nominal environmental situations.

## 4 Materials and Methods

### 4.1 Multiobjective Optimization

Generally, a multiobjective problem is faced by building an aggregate function in order to assemble the objectives in a unique index that contains a weighting vector for each objective. However, the solution obtained is determined by the selection of the weighting values. An alternative option is to use a multiobjective optimization design (Miettinen, 1999). In multiobjective optimization all objectives are important, therefore all of them are optimized simultaneously. Instead of one rarely unique solution, we obtain a set of the best solutions known as *Pareto Front*. In this front, all solutions are *Pareto-optimal* and only differ from each other in the trade-off of objectives each one represents. Multiobjective optimization requires at least three fundamental steps (Miettinen et al., 2008): 1) the multiobjective problem definition (MOP), 2) the optimization process, and 3) the multicriteria decision making process (MCDM). The overall multiobjective optimization design enables us to analyze current trade-offs between the objectives and select the most suitable solutions (Reynoso-Meza et al., 2013) that reaches all of our objectives.

### 4.2 Multiobjective Problem Definition

As referred in (Miettinen et al., 2008), a Multiobjective Problem (MOP), can be stated as follows:

$$\begin{array}{ll} \underset{\theta }{\min}J\left(\theta \right)= & \left[J_{1}\left(\theta \right),\ldots ,J_{m}\left(\theta \right)\right]\\ subject\;to:\; & K\left(\theta \right)\leq 0\\ & \underline{\theta_{i}}\leq \theta_{i}\leq \bar{\theta_{i}},\;\;\forall i=\left[1,\ldots ,n\right] \end{array}$$ (19)

Where ***θ*** = [*θ*_1_, *θ*_2_, …, *θ*_*n*_] is the decision vector that contains the decision variables for multiobjective optimization; **J**(***θ***) is the objective vector and **K**(***θ***), **L**(***θ***) are the inequality and equality constraint vectors, respectively, $\underline{\theta_{i}}$ , $\bar{\theta_{i}}$ are the lower and upper bounds in the decision variables space **Θ**. The MOP (4.2) has a set of solutions whose values in the Pareto front are function of the decision variables defined as the Pareto Set **Θ**_***P***_. Each solution in this set corresponds to an optimal objective vector in the Pareto Front **J**_**P**_. All solutions in the the Pareto Set are Pareto-optimal non dominated solutions, that is, they differ from each other in the objectives trade-off each one represents.

### 4.3 Multiobjective Problem of the Merging Pathway

Here, the objective vector **J**(***θ***) has to be defined to solve the problem presented in 2.1. We maximized the desired target titer for product *P* while minimizing the perturbation effects on the substrate *S*_2_ dynamics. The objective functions **J**_**1**_ and **J**_**2**_ were defined before in Eqs. 9, 10, and the decision variables ***θ*** used for our optimization are ***θ*** = [a_1_, k_d20_, k_a*σ*_, *μ*] with their corresponding lower and upper bounds as detailed in Table 1.

**TABLE 1:** Lower and upper bounds for the merging pathway MOP.

Bound	k_a*σ*_ (min^−1^)	k_d20_ (*molec*)	a_1_ (min^−1^)	*μ* (min^−1^)
Lower	700	100,000	90	0.005
Upper	1,500	350,000	160	0.01

Hence, the MOP in (4.2) can be stated as:

$$\begin{array}{ll} \underset{\theta \in R^{4}}{\min}J\left(\theta \right)= & \left[J_{1}\left(\theta \right),J_{2}\left(\theta \right)\right]\in R^{2}\\ \text{subject to: } & equations\;\left(1-8\right) \end{array}$$ (20)

### 4.4 Naringenin Pathway as a Multiobjective Problem

For the dynamic regulation of the naringenin pathway, we defined three objective functions to tune both antithetic controller together with the biosensor:

$$J_{1}=\left|Target-K_{Nar}N_{\text{unp}}\left(T\right)\right|,\;\;\;\left(\text{mg/L target titer error}\right)$$ (21)

$$J_{2}=\left|\frac{N_{\text{unp}}\left(T\right)-N_{\text{pert}}\left(T\right)}{N_{\text{unp}}\left(T\right)}\right|,\left(\text{\% production loss after perturbation}\right)$$ (22)

$$J_{3}=\frac{1}{2}\overset{N}{\underset{k=1}{\sum }}{\left(X\left(k\right)-X\left(k-1\right)\right)}^{2},\;\;\;\left(\text{number of oscillations}\right)$$ (23)

Where $K_{Nar}=\frac{mw\cdot x\left(T\right)}{A_{\text{v}}}$ is a constant that converts amounts of naringenin into grams per liter the titer units, mw = 272.25 (g/mol) is the naringenin’s molecular weight, A_v_ is the Avogadro’s number, *x*(*T*) is the number of cells at time T, and $X\left(k\right)=\frac{2\;\mathrm{sign}\left.\left(\left(mean\left(\sigma_{\text{unp}}\left(k\right)\right)-\sigma_{\text{unp}}\left(k\right)\right)+1\right)\right)}{2}$ is the clipped binary version of *σ* factor used to detect its zero-crossing and obtain the number of oscillations of the *σ* before the malonyl perturbation at the position *k*. Additionally, the constraints vector **K**(***θ***) set two significant limitations for the antithetic controller performance in this pathway:

$$\left[\sigma \right]\geq 4.5\mu M$$ (24)

σ≥aσ, (25)

As we said in Section 2.3, seven decision variables from the biocontroller and biosensor kinetics in Eqs. 13, 14 were selected. Particularly, we considered the ones that are prone to be modified in the wet-lab:

$$\theta =\left[p_{\text{a}\sigma },\;C_{{\text{N}}_{\text{a}\sigma }},\;p_{\text{h}},\;C_{{\text{N}}_{\text{h}}},\;k_{\text{c}\sigma },\;k_{\text{d}20},\;\mu \right],$$ (26)

Table 2 defines the lower and upper limits of the parameters selected for tuning within standard ranges for the chosen biological parts. Therefore, altogether can state the MOP of the naringenin pathway as follows:

$$\begin{array}{ll} \underset{\theta \in R^{7}}{\min}J\left(\theta \right)= & \left[J_{1}\left(\theta \right),J_{2}\left(\theta \right),J_{3}\left(\theta \right)\right]\in R^{3}\\ \text{subject to: } & equations\;\left(11\right)-\left(15\right)\\ & constraints\;\left(24\right)-\left(25\right) \end{array}$$ (27)

**TABLE 2:** Lower and upper bounds for the naringenin pathway MOP.

Bound	p_a_	C_Na_	p_h_	C_Nh_	k_c_	k_d20_	*μ*
	**(min^−1^)**	**(copies)**	**(min^−1^)**	**(copies)**	**(min^−1^)**	**(molec)**	**(min^−1^)**
Lower	0.1	1	0.1	1	0.01	1*e* − 2	0.006 9
Upper	20	15	20	15	20	1*e*4	0.023 1

For the rest of the parameters, a sensitivity analisys was performed in previous work. Evidently, using different values of the these parameters may have an impact on the resulting behavior of the system, however this does not invalidate the whole methodology we are presenting here.

### 4.5 Multiobjective Optimization Process

The multiobjective optimization process finds the best parameters ${\theta_{P}}^{*}$ producing the best Pareto front approximation $J_{P}{\left(\theta_{P}\right)}^{*}$ for each MOP. Evolutionary algorithms are one of the suggested optimization techniques to address optimization problems generally present in systems and synthetic biology (Moles et al., 2003). We used a multiobjective evolutionary algorithm based on differential evolution, which uses a spherical pruning to approximate the Pareto front. The implementation comes from the sp-MODEx^1^ algorithm that improves: 1) convergence by using an external file to store solutions and include them in the evolutionary process, 2) spreading by using the spherical pruning mechanism, and 3) pertinency of solutions *via* a basic bound mechanism in the objective space (Reynoso-Meza et al., 2014).

#### 4.5.1 Multicriteria Decision Making Process

Choosing the preferable solution according to designer’s criteria takes place in an *a-posteriori* multicriteria decision making stage of the Pareto front obtained. It is desirable to have tools that simplify the visualization as well as the analysis of the trade-off among competing objectives. This could be a non-trivial task when the number of objectives is larger than three and/or the number of decision variables in the Pareto set is large enough, like in our case. We used the *Level Diagrams Toolbox* (Blasco et al., 2017) from Matlab (LD-Tool^2^) as the Pareto front visualization tool, which is freely available for designers. LD-Tool correlates the design objectives **J**_**P**_(***θ***) with their decision variables ***θ*** by illustrating two graphs. The first graph contains each objective, where its *Y*-axis is the p-norm ‖*J*(***θ***)‖_*p*_ of the objectives vector, and the *X*-axis corresponds to each objective value *J*_*i*_(***θ***). The second graph shows ‖*J*(***θ***)‖_*p*_ with respect to every ***θ***, so a given solution will have the same *y*-value in all graphs.

### 4.6 Computational Simulations

All simulations of both the merging metabolic pathway and the naringenin pathway were performed in Matlab, using a 4 Core processor, 16 GB RAM *@* 3.80 GHz. First, we defined two sets of model parameters known as nominal parameters for each system. Then, we computed the number of molecules of each species from every *i* − cell in the population over time. These data allow us to obtain the performance, robustness and stability of the biosensor and the antithetic controller from each system. Finally, we tuned the biosensor and the biocontroller for optimal dynamic pathway regulation following the multiobjective approach. For the merging metabolic pathway, the sp-MODEx evolutionary algorithm evaluated 1,000 the cost function, using 125 generations and taking 1 h for a simulation time. For the naringenin pathway dynamic regulation, the sp-MODEx evaluated 10,000 times the cost function, using 199 generations over 8 h of simulation time.

All the scripts of the simulations and optimization can be found in the Github repository https://github.com/sb2cl/molecular-biocontroller-tuning.

## Data Availability

The datasets generated and analyzed for this study can be found in the Github repository https://github.com/sb2cl/molecular-biocontroller-tuning.

## Footnotes

1 Available in http://www.mathworks.com/matlabcentral/fileexchange/65145

2 Available at http://www.mathworks.com/matlabcentral/fileexchange/24042

## References

[B1] Aoki S. K. Lillacci G. Gupta A. Baumschlager A. Schweingruber D. Khammash M. (2019). A Universal Biomolecular Integral Feedback Controller for Robust Perfect Adaptation. Nature 570, 533–537. 10.1038/s41586-019-1321-1 31217585

[B2] Blair R. H. Kliebenstein D. J. Churchill G. A. (2012). What Can Causal Networks Tell Us about Metabolic Pathways? Plos Comput. Biol. 8, e1002458. 10.1371/journal.pcbi.1002458 22496633 PMC3320578

[B3] Blasco X. Herrero J. M. Reynoso-Meza G. Martínez Iranzo M. A. (2017). “Interactive Tool for Analyzing Multiobjective Optimization Results with Level Diagrams,” in Proceedings of the Genetic and Evolutionary Computation Conference Companion, Berlin, Germany, July 15–19, 2017, 1689–1696. 10.1145/3067695.3082553

[B4] Boada Y. Picó J. Vignoni A. (2021). “Multi-objective Optimization Tuning Framework for Kinetic Parameter Selection and Estimation,” in Methods in Molecular Biology (New York, NY: Springer US), 2385. 10.1007/978-1-0716-1767-0) 34888716

[B5] Boada Y. Reynoso-Meza G. Picó J. Vignoni A. (2016). Multi-objective Optimization Framework to Obtain Model-Based Guidelines for Tuning Biological Synthetic Devices: An Adaptive Network Case. BMC Syst. Biol. 10, 27. 10.1186/s12918-016-0269-0 26968941 PMC4788947

[B6] Boada Y. Vignoni A. Alarcon‐Ruiz I. Andreu‐Vilarroig C. Monfort‐Llorens R. Requena A. (2019a). Characterization of Gene Circuit Parts Based on Multiobjective Optimization by Using Standard Calibrated Measurements. ChemBioChem 20, 2653–2665. 10.1002/cbic.201900272 31269324

[B7] Boada Y. Vignoni A. Picó J. Carbonell P. (2020). Extended Metabolic Biosensor Design for Dynamic Pathway Regulation of Cell Factories. iScience 23, 101305. 10.1016/j.isci.2020.101305 32629420 PMC7334618

[B8] Boada Y. Vignoni A. Picó J. (2017a). Engineered Control of Genetic Variability Reveals Interplay Among Quorum Sensing, Feedback Regulation, and Biochemical Noise. ACS Synth. Biol. 6, 1903–1912. 10.1021/acssynbio.7b00087 28581725

[B9] Boada Y. Vignoni A. Picó J. (2017b). Multi-Objective Optimization for Gene Expression Noise Reduction in a Synthetic Gene Circuit. IFAC-PapersOnLine 50, 4472–4477. 10.1016/j.ifacol.2017.08.376

[B10] Boada Y. Vignoni A. Pico J. (2019b). Multiobjective Identification of a Feedback Synthetic Gene Circuit. IEEE Trans. Contr. Syst. Technol. 28, 208–223. 10.1109/TCST.2018.2885694

[B11] Briat C. Gupta A. Khammash M. (2016). Antithetic Integral Feedback Ensures Robust Perfect Adaptation in Noisy Biomolecular Networks. Cel Syst. 2, 15–26. 10.1016/j.cels.2016.01.004 27136686

[B26] [Dataset] Otero-Muras I. Carbonell P. (2021). Automated Engineering of Synthetic Metabolic Pathways for Efficient Biomanufacturing. Metab. Eng. 63, 61–80. 10.1016/j.ymben.2020.11.012 33316374

[B12] Dunstan M. S. Robinson C. J. Jervis A. J. Yan C. Carbonell P. Hollywood K. A. (2020). Engineering *Escherichia coli* towards De Novo Production of Gatekeeper (2S)-Flavanones: Naringenin, Pinocembrin, Eriodictyol and Homoeriodictyol. Synth. Biol. (Oxf) 5, ysaa012. 10.1093/synbio/ysaa012 33195815 PMC7644443

[B13] Gao C. Xu P. Ye C. Chen X. Liu L. (2019). Genetic Circuit-Assisted Smart Microbial Engineering. Trends Microbiol. 27, 1011–1024. 10.1016/j.tim.2019.07.005 31421969

[B14] Hartline C. J. Schmitz A. C. Han Y. Zhang F. (2020). Dynamic Control in Metabolic Engineering: Theories, Tools, and Applications. Metab. Eng. 63, 126–140. 10.1016/j.ymben.2020.08.015 32927059 PMC8015268

[B15] Johnson A. O. Gonzalez-Villanueva M. Wong L. Steinbüchel A. Tee K. L. Xu P. (2017). Design and Application of Genetically-Encoded Malonyl-CoA Biosensors for Metabolic Engineering of Microbial Cell Factories. Metab. Eng. 44, 253–264. 10.1016/j.ymben.2017.10.011 29097310

[B16] Li G. Deng L. Xiao G. Tang P. Wen C. Hu W. (2018). Enabling Controlling Complex Networks with Local Topological Information. Sci. Rep. 8, 4593. 10.1038/s41598-018-22655-5 29545560 PMC5854593

[B17] Liu D. Mannan A. A. Han Y. Oyarzún D. A. Zhang F. (2018). Dynamic Metabolic Control: Towards Precision Engineering of Metabolism. J. Ind. Microbiol. Biotechnol. 45, 535–543. 10.1007/s10295-018-2013-9 29380150

[B18] Liu D. Zhang F. (2018). Metabolic Feedback Circuits Provide Rapid Control of Metabolite Dynamics. ACS Synth. Biol. 7, 347–356. 10.1021/acssynbio.7b00342 29298043

[B19] Liu L. Shan S. Zhang K. Ning Z.-Q. Lu X.-P. Cheng Y.-Y. (2008). Naringenin and Hesperetin, Two Flavonoids Derived fromCitrus Aurantiumup-Regulate Transcription of Adiponectin. Phytother. Res. 22, 1400–1403. 10.1002/ptr.2504 18690615

[B20] Lo-Thong O. Charton P. Cadet X. F. Grondin-Perez B. Saavedra E. Damour C. (2020). Identification of Flux Checkpoints in a Metabolic Pathway through White-Box, Grey-Box and Black-Box Modeling Approaches. Sci. Rep. 10, 13446. 10.1038/s41598-020-70295-5 32778715 PMC7417601

[B21] Michaelis L. Menten M. (1913). Die kinetik der invertinwirkung biochem z. Biochem. z. Berlin: Berlin, 49, 333–369. Find this article online.

[B22] Miettinen K. (1999). Nonlinear Multiobjective Optimization, 12. Boston: Kluwer Academic Publishers.

[B23] Miettinen K. Ruiz F. Wierzbicki A. P. (2008). “Introduction to Multiobjective Optimization: Interactive Approaches,” in Multiobjective Optimization. Editors Branke J. Deb K. Miettinen K. Słowiński R (Berlin, Heidelberg: Springer), 27–57. 10.1007/978-3-540-88908-3_2

[B24] Moles C. G. Mendes P. Banga J. R. (2003). Parameter Estimation in Biochemical Pathways: A Comparison of Global Optimization Methods. Genome Res. 13, 2467–2474. 10.1101/gr.1262503 14559783 PMC403766

[B25] Otero-Muras I. Banga J. R. (2017). Automated Design Framework for Synthetic Biology Exploiting Pareto Optimality. ACS Synth. Biol. 6, 1180–1193. 10.1021/acssynbio.6b00306 28350462

[B27] Oyarzún D. A. Stan G.-B. V. (2013). Synthetic Gene Circuits for Metabolic Control: Design Trade-Offs and Constraints. J. R. Soc. Interf. 10, 20120671. 10.1098/rsif.2012.0671 PMC3565798 23054953

[B28] Rahigude A. Bhutada P. Kaulaskar S. Aswar M. Otari K. (2012). Participation of Antioxidant and Cholinergic System in Protective Effect of Naringenin against Type-2 Diabetes-Induced Memory Dysfunction in Rats. Neuroscience 226, 62–72. 10.1016/j.neuroscience.2012.09.026 22999973

[B29] Reynoso-Meza G. Garcia-Nieto S. Sanchis J. Blasco F. X. (2013). Controller Tuning Using Multiobjective Optimization Algorithms: a Global Tuning Framework. IEEE Trans. Control. Syst. Technol. 21, 445–458. 10.1109/tcst.2012.2185698

[B30] Reynoso-Meza G. Sanchis J. Blasco X. García-Nieto S. (2014). Physical Programming for Preference Driven Evolutionary Multi-Objective Optimization. Appl. Soft Comput. 24, 341–362. 10.1016/j.asoc.2014.07.009

[B31] Santos-Navarro F. N. Boada Y. Vignoni A. Picó J. (2021). Gene Expression Space Shapes the Bioprocess Trade-Offs Among Titer, Yield and Productivity. Appl. Sci. 11, 5859. 10.3390/app11135859

[B32] Schomburg I. Jeske L. Ulbrich M. Placzek S. Chang A. Schomburg D. (2017). The BRENDA Enzyme Information System-From a Database to an Expert System. J. Biotechnol. 261, 194–206. 10.1016/j.jbiotec.2017.04.020 28438579

[B33] Segall-Shapiro T. H. Sontag E. D. Voigt C. A. (2018). Engineered Promoters Enable Constant Gene Expression at Any Copy Number in Bacteria. Nat. Biotechnol. 36, 352–358. 10.1038/nbt.4111 29553576

[B34] Sheng H. Sun X. Yan Y. Yuan Q. Wang J. Shen X. (2020). Metabolic Engineering of Microorganisms for the Production of Flavonoids. Front. Bioeng. Biotechnol. 8, 1–15. 10.3389/fbioe.2020.589069 33117787 PMC7576676

[B35] Siedler S. Stahlhut S. G. Malla S. Maury J. Neves A. R. (2014). Novel Biosensors Based on Flavonoid-Responsive Transcriptional Regulators Introduced into escherichia Coli. Metab. Eng. 21, 2–8. 10.1016/j.ymben.2013.10.011 24188962

[B36] Stevens J. T. Carothers J. M. (2015). Designing RNA-Based Genetic Control Systems for Efficient Production from Engineered Metabolic Pathways. ACS Synth. Biol. 4, 107–115. 10.1021/sb400201u 25314371

[B37] Takamura Y. Nomura G. (1988). Changes in the Intracellular Concentration of Acetyl-CoA and Malonyl-CoA in Relation to the Carbon and Energy Metabolism of escherichia Coli K12. Microbiology 134, 2249–2253. 10.1099/00221287-134-8-2249 3075658

[B38] Tsiantis N. Banga J. R. (2020). Using Optimal Control to Understand Complex Metabolic Pathways. BMC Bioinformatics 21, 472. 10.1186/s12859-020-03808-8 33087041 PMC7579911

[B39] Wehrs M. Tanjore D. Eng T. Lievense J. Pray T. R. Mukhopadhyay A. (2019). Engineering Robust Production Microbes for Large-Scale Cultivation. Trends Microbiol. 27, 524–537. 10.1016/j.tim.2019.01.006 30819548

[B40] Wu J. Du G. Chen J. Zhou J. (2015). Enhancing Flavonoid Production by Systematically Tuning the Central Metabolic Pathways Based on a CRISPR Interference System in escherichia Coli. Sci. Rep. 5, 13477. 10.1038/srep13477 26323217 PMC4555050

[B41] Xu P. Li L. Zhang F. Stephanopoulos G. Koffas M. (2014). Improving Fatty Acids Production by Engineering Dynamic Pathway Regulation and Metabolic Control. Proc. Natl. Acad. Sci. 111, 11299–11304. 10.1073/pnas.1406401111 25049420 PMC4128127

[B42] Yang L. Ebrahim A. Lloyd C. J. Saunders M. A. Palsson B. O. (2019). DynamicME: Dynamic Simulation and Refinement of Integrated Models of Metabolism and Protein Expression. BMC Syst. Biol. 13, 2. 10.1186/s12918-018-0675-6 30626386 PMC6327497

[B43] Yang Y. Lin Y. Li L. Linhardt R. J. Yan Y. (2015). Regulating Malonyl-CoA Metabolism via Synthetic Antisense RNAs for Enhanced Biosynthesis of Natural Products. Metab. Eng. 29, 217–226. 10.1016/j.ymben.2015.03.018 25863265

[B44] Zygmunt K. Faubert B. MacNeil J. Tsiani E. (2010). Naringenin, a Citrus Flavonoid, Increases Muscle Cell Glucose Uptake via AMPK. Biochem. Biophysical Res. Commun. 398, 178–183. 10.1016/j.bbrc.2010.06.048 20558145

