# Supplementary material for: Modeling and Optimization of a Molecular Biocontroller for the Regulation of Complex Metabolic Pathways
Source: Front Mol Biosci. 2022 Mar 29;9:801032. doi: 10.3389/fmolb.2022.801032 (PMC9001882; doi:10.3389/fmolb.2022.801032)
Supplement: Supplementary file 1 [file DataSheet1.PDF]

Supplementary Material

1 DYNAMIC REGULATION OF THE MERGING METABOLIC PATHWAY

1.1 Pareto solutions

Solution	J_1	J_2	$\mathrm{k_{a\sigma}}$ ($\mathrm{min^{-1}}$)	$\mathrm{k_{d20}}$ (molec)	$\mathrm{a_1}$ ($\mathrm{min^{-1}}$)	μ ($\mathrm{min^{-1}}$)
1	469.7717	0.0046213	1400	100000	154	0.00985
2	361.7292	0.0060863	1150	150000	142	0.0092
3	268.2432	0.020826	800	250000	110	0.0087
4	250.4551	0.055449	900	300000	116	0.0086
5	245.3615	0.083188	1000	350000	124	0.0085

Table S1. Pareto Solution. Library of biosensors-controller for the merging metabolic pathway.

1.2 Model parameters

Parameter	Description	Value	Unit	Reference
a_0	E constitutive expression	0.154	$\mathrm{min^{-1}}$	estimated
TF	Biosensor TF concentration	0.154	molec	estimated
$\mathrm{C_N}$, $\mathrm{C_{Na\sigma}}$	Plasmid copy number	10	copies	(Boada et al., 2017)
$\mathrm{k_\sigma}$	Expression strength	10	$\mathrm{min^{-1}}$	estimated
$\mathrm{d_c}$	degradation rate $[\sigma \cdot a\sigma]$	1×10^{-3}	$\mathrm{min^{-1}}$	(Annunziata et al., 2017)
$\mathrm{kd_p}$	dissociation constant TF to P	1.5×10^4	molec	estimated
γ	dissociation constant $[\sigma \cdot a\sigma]$	0.01	$\mathrm{molec^{-1}\ min^{-1}}$	(Buchler et al., 2005)
$\mathrm{d_\sigma}$, $\mathrm{d_{a\sigma}}$, $\mathrm{d_E}$	protein degradation rate	3×10^{-4}	$\mathrm{min^{-1}}$	typical lifetime=38 h
$\mathrm{k_{cat}}$	F3H catalytic constant	0.42	$\mathrm{min^{-1}}$	BRENDA:EC 1.14.11.11
$\mathrm{K_{mS_1}}$	Michaelis constant $\mathrm{E - S_2}$	1e-3	molec	estimated
$\mathrm{K_{mS_2}}$	Michaelis constant $\mathrm{E - S_1}$	1e-3	molec	estimated

Table S2. Model parameters of the merging metabolic pathway model.

1.3 Temporal responses

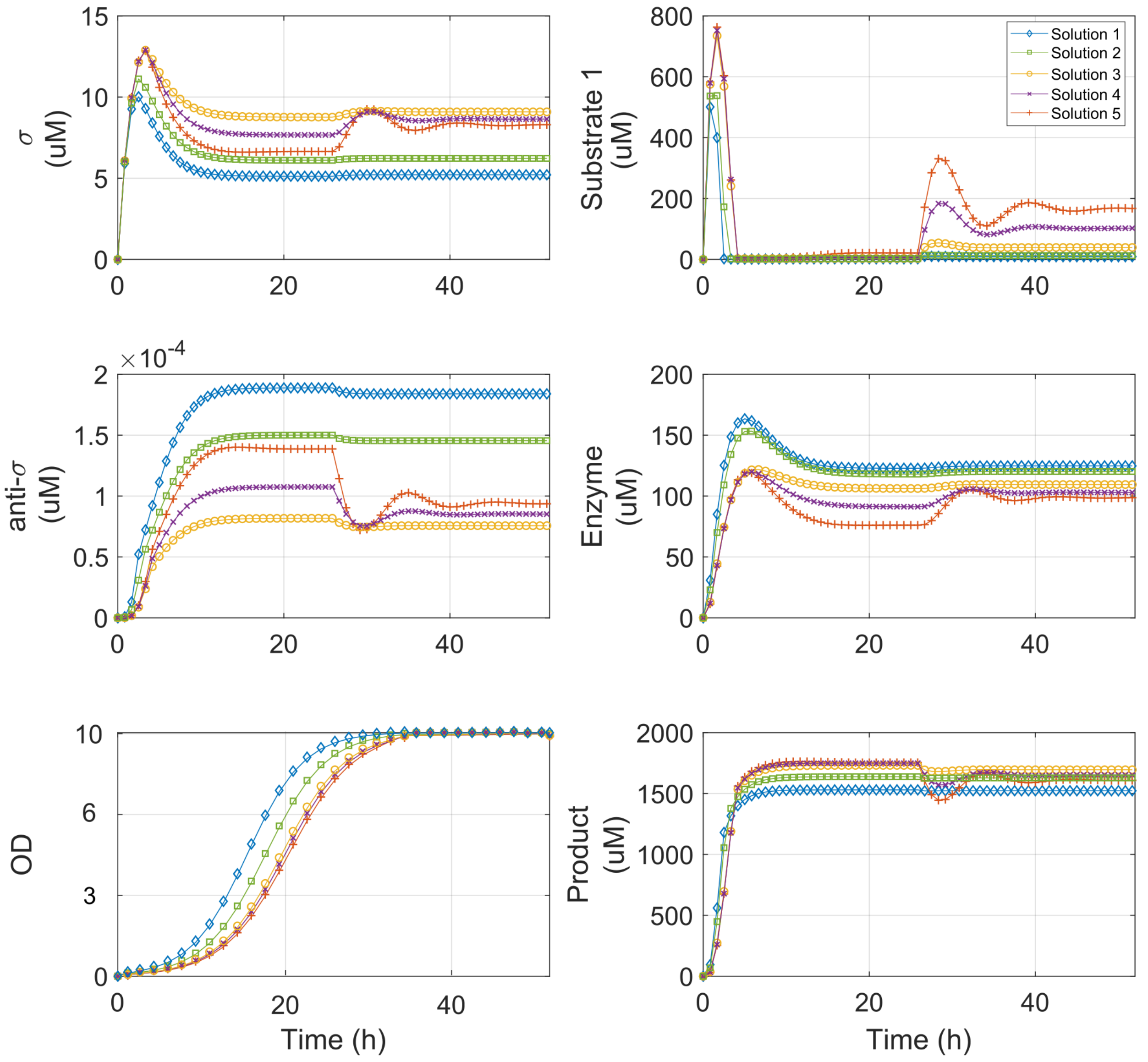

Figure S1. Temporal responses of the selected solutions

ó

2 NARINGENIN'S PATHWAY OPTIMIZATION AND DYNAMIC REGULATION

2.1 Pareto solutions

Solution	J_1	J_2	J_3	p_a (min^{-1})	C_{Na} (copies)	p_h (min^{-1})	C_{Nh} (copies)	k_c (min^{-1})	k_{d20} (molec)	μ (min^{-1})
1	0.02	38.58	1.00	10.00	3.33	9.87	8.68	0.01	10000.00	0.0069
2	0.20	39.34	0.50	2.77	6.38	14.74	13.33	10.59	2929.26	0.0104
3	0.35	34.54	1.00	4.94	5.32	20.00	15.00	17.98	10000.00	0.0106
4	0.67	26.87	1.00	3.36	8.53	10.76	13.93	4.67	10000.00	0.0069
5	0.73	22.17	2.50	10.00	4.68	9.45	15.00	0.01	8229.50	0.0069
6	0.76	24.17	2.00	9.88	4.79	15.68	11.83	20.00	7307.36	0.0081
7	0.87	22.88	2.00	5.18	6.66	11.47	15.00	0.01	10000.00	0.0071
8	2.20	16.62	2.50	10.00	4.84	13.89	13.33	11.60	10000.00	0.0069
9	2.92	38.20	0.50	1.53	10.60	20.00	7.29	8.30	9507.13	0.0085
10	7.78	21.36	2.00	5.32	6.66	16.53	15.00	3.59	8891.73	0.0082
11	8.36	10.22	2.50	10.00	4.88	15.04	14.60	0.01	9898.57	0.0069
12	14.08	9.64	2.50	10.00	4.88	15.04	15.00	0.01	9898.57	0.0070
13	20.08	26.44	1.00	3.36	8.53	10.76	14.92	20.00	10000.00	0.0069
14	76.21	8.86	2.50	10.00	5.01	13.89	15.00	0.01	9753.57	0.0069
15	119.90	8.48	2.50	10.00	5.10	13.33	15.00	0.01	10000.00	0.0069

Table S3. Pareto Solution. Library of biosensors-controller for the naringenin pathway dynamic regulation.

2.2 Pareto front and Pareto set

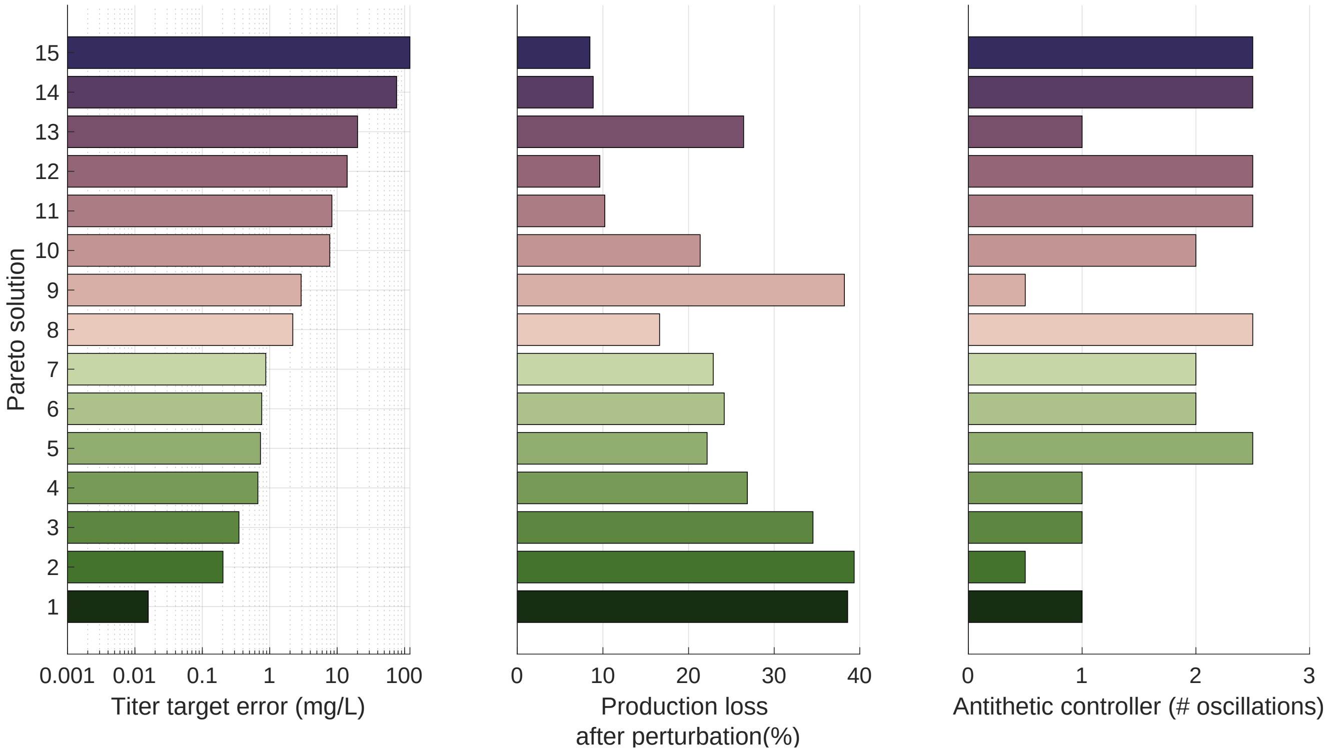

Figure S2. Objectives $\mathbf{J_1}$, $\mathbf{J_2}$ and $\mathbf{J_3}$ from the naringenin Pareto front.

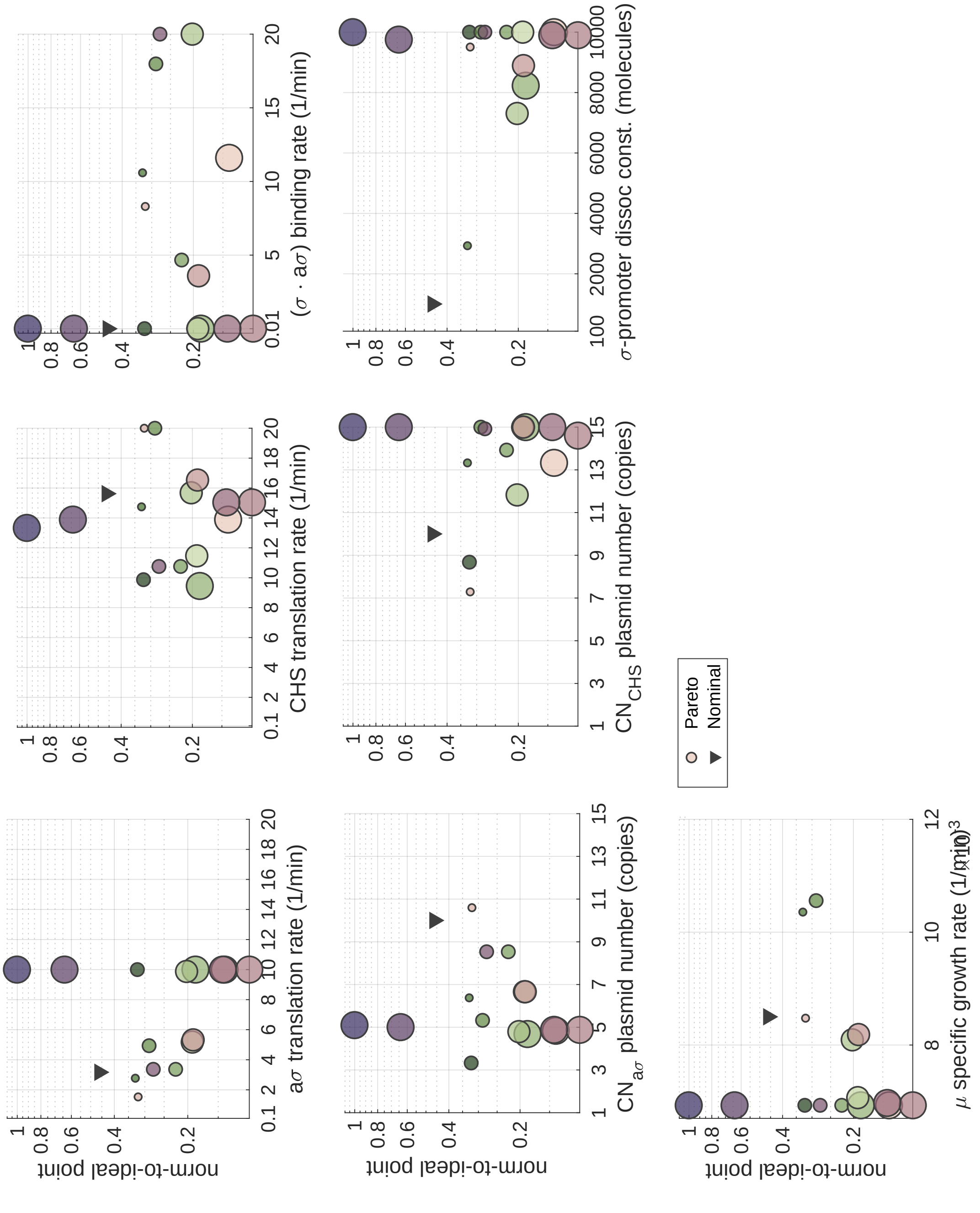

Figure S3. Level Diagram Pareto set containing the parameter combinations from multiobjective optimization
.

2.3 Model parameters

Parameter	Description	Value	Unit
TAL (EC 4.3.1.23)*	Enzyme amount	3.2×10^5	molec
4CL (EC 6.2.1.12)	Enzyme amount	6.48×10^5	molec
CHI (EC 5.5.1.6)	Enzyme amount	3.54×10^5	molec
F3H (EC 1.14.11.11)	Enzyme amount	2.81	molec
FLS (EC 1.14.20.6)	Enzyme amount	5.84	molec
CHS (EC 2.3.1.74)	Basal (open loop) enzyme amount	2.13×10^5	molec
M_a (BRENDA ID 13046)	Malonyl-CoA initial amount	2.34×10^4	molec
K_{Lt} **	L-tyrosine flux	2×10^6	$\mathrm{molec} \cdot \mathrm{min}^{-1}$
k_{catTAL}	TAL catalytic constant	1.2	min^{-1}
k_{cat4CL}	4CL catalytic constant	0.492	min^{-1}
k_{catCHS}	CHS catalytic constant	1.68	min^{-1}
k_{catCHI}	CHI catalytic constant	4.2	min^{-1}
k_{catF3H}	F3H catalytic constant	174	min^{-1}
k_{catFLS}	FLS catalytic constant	6	min^{-1}
K_{mLt}	Michaelis constant $\mathrm{TAL} - \mathrm{Lt}$	1.9×10^4	molec
K_{mpC}	Michaelis constant $\mathrm{4CL} - \mathrm{pC}$	1.4×10^4	molec
K_{mMa}	constant $\mathrm{CHS} - \mathrm{M}$	1×10^{-3}	molec
K_{mpA}	constant $\mathrm{CHS} - \mathrm{pA}$	1×10^{-3}	molec
K_{mNc}	Michaelis constant $\mathrm{CHI} - \mathrm{Nc}$	2.8×10^4	molec
K_{mN}	Michaelis constant $\mathrm{F3H} - \mathrm{N}$	5×10^8	molec
K_{mDi}	Michaelis constant $\mathrm{FHS} - \mathrm{Di}$	1×10^4	molec

Table S4. Table S4. Model parameters of the naringenin pathway model.

* EC refers to the corresponding BRENDA:EC identification number (Schomburg et al., 2017).
** This flux is equivalent to add 3 mM of L-tyrosine in a batch bioreactor with an initial OD_{600}=0.001.

Parameter	Description	Value	Unit	Reference
α	tight basal expression	0.01	adim	estimated
β	constitutive expression	1.5	adim	estimated
D	AHL diffusion rate across cell membrane	2	min^{-1}	(Boada et al., 2017)
V_{ext}	bioreactor volume	4×10^{-3}	L	-
V_{cell}	cell volume	1.1×10^{-15}	L	(Milo et al., 2010)
$x^{\flat}_{max}$	maximum growth capacity	3.84×10^{11}	cells	(Milo et al., 2010)
C_N	plasmid copy number	10	copies	typical small copy number
k_σ, $k_a\sigma$	σ, $a\sigma$ transcription rate	1.98	min^{-1}	estimated
k_R	*luxR* transcription rate	0.78	min^{-1}	optimised♣
k_H	*chs* transcription rate	3.67	min^{-1}	optimised♣
k_Q	*qdoR* transcription rate	0.71	min^{-1}	optimised♣
p_σ	σ translation rate	3	min^{-1}	optimised$^\diamondsuit$
p_R	LuxR translation rate	2.34	min^{-1}	optimised$^\diamondsuit$
p_{Hc}	CHS constitutive translation rate	6.5×10^{-4}	min^{-1}	estimated
p_Q	QdoR translation rate	2.55	min^{-1}	optimised$^\diamondsuit$
d_R	LuxR degradation rate	0.02	min^{-1}	(Boada et al., 2017) and refs. therein
d_c	$[\sigma \cdot a\sigma]$ degradation rate	1×10^{-3}	min^{-1}	(Annunziata et al., 2017)
d_A	Intracellular AHL degradation rate	4×10^{-4}	min^{-1}	(Kaufmann et al., 2005)
d_{Ae}	Extracellular AHL degradation rate	4.8×10^{-5}	min^{-1}	(Fekete et al., 2007; Boada et al., 2020)
kd_{20}	dissociation constant to promoter p_{20}	1000	molec	(Aoki et al., 2019)
kd_{lux}	dissociation constant to promoter p_{lux}	600	molec	Boada et al. (2017); Buchler et al. (2005)
kd_σ	dissociation constant σ dimer	1000	molec	estimated
kd_q	dissociation constant to promoter p_{qdoI}	150	molec	(Boada et al., 2020; Siedler et al., 2014)
kd_k	dissociation constant QdoR to kaempferol	75	molec	(Siedler et al., 2014)
kd_c	dissociation constant $[\sigma \cdot a\sigma]$	0.01	molec	(Annunziata et al., 2017)
dm_σ, $dm_{a\sigma}$	mRNA degradation rate	0.231	min^{-1}	typical lifetime= 3 min
dm_R, dm_H, dm_Q	mRNA degradation rate	0.231	min^{-1}	typical lifetime= 3 min
d_σ, $d_{a\sigma}$, d_H, d_Q	protein degradation rate	3×10^{-4}	min^{-1}	typical lifetime= 3 min

Table S5. Table S5. Model parameters of the biosensor and antithetic controller model.

$^\flat$ This value is equivalent to an OD_{600}=120 at the end of the experiment.

♣ Each mRNA transcriptional rate is an average rate obtained as the product of the coding sequence length (base pairs) and the minimum and maximum effective rates (min^{-1}) of the RNA polimerase to transcribe a gene in *E. coli* (Buchler et al., 2005; Alon, 2019).

$\diamondsuit$ Each translation rate is an average rate obtained as the product of the coding sequence length (base pairs), the minimum and maximum

effective translation rates (min^{-1}) of a ribosome along a mRNA molecule, and the ribosomal density for that mRNA molecule in *E. coli* (Alon, 2019).

2.4 Temporal responses

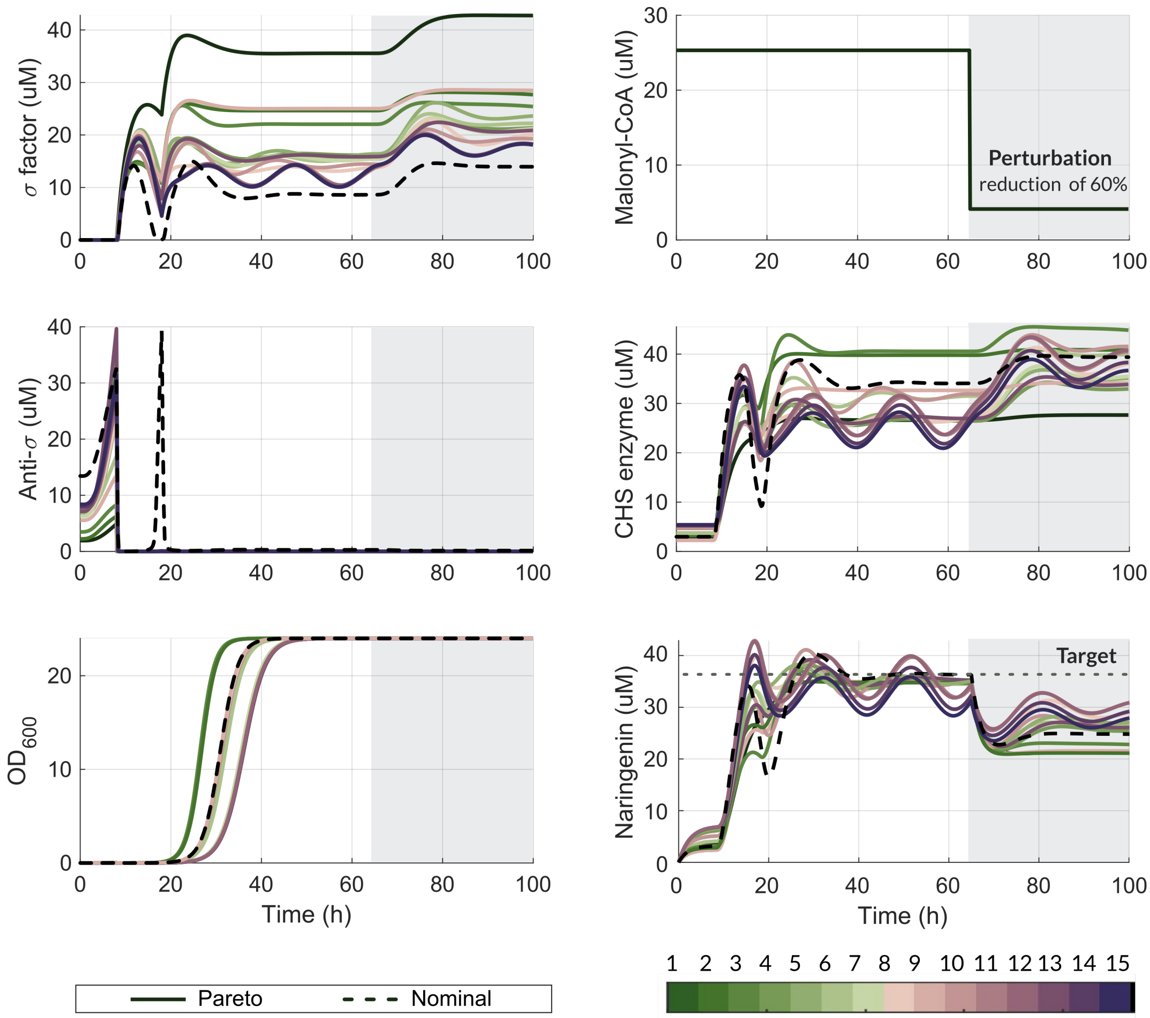

Figure S4. Temporal responses of the solutions for the naringenin pathway dynamic regulation tuning.

REFERENCES

Alon, U. (2019). *An introduction to systems biology: design principles of biological circuits* (CRC press)

Annunziata, F., Matyjaszkiewicz, A., Fiore, G., Grierson, C. S., Marucci, L., di Bernardo, M., et al. (2017). An orthogonal multi-input integration system to control gene expression in escherichia coli. *ACS Synthetic Biology* 6, 1816–1824. doi:10.1021/acssynbio.7b00109

Aoki, S. K., Lillacci, G., Gupta, A., Baumschlager, A., Schweingruber, D., and Khammash, M. (2019). A universal biomolecular integral feedback controller for robust perfect adaptation. *Nature* 570, 533–537. doi:10.1038/s41586-019-1321-1

Boada, Y., Vignoni, A., and Picó, J. (2017). Engineered control of genetic variability reveals interplay among quorum sensing, feedback regulation, and biochemical noise. *ACS synthetic biology* 6, 1903–1912

Boada, Y., Vignoni, A., Picó, J., and Carbonell, P. (2020). Extended Metabolic Biosensor Design for Dynamic Pathway Regulation of Cell Factories. *iScience* 23. doi:10.1016/j.isci.2020.101305

Buchler, N. E., Gerland, U., and Hwa, T. (2005). Nonlinear protein degradation and the function of genetic circuits. *Proceedings of the National Academy of Sciences* 102, 9559–9564

Fekete, A., Frommberger, M., Rothballer, M., Li, X., Englmann, M., Fekete, J., et al. (2007). Identification of bacterial n-acylhomoserine lactones (ahls) with a combination of ultra-performance liquid chromatography (uplc), ultra-high-resolution mass spectrometry, and in-situ biosensors. *Analytical and bioanalytical chemistry* 387, 455–467

Kaufmann, G. F., Sartorio, R., Lee, S.-H., Rogers, C. J., Meijler, M. M., Moss, J. A., et al. (2005). Revisiting quorum sensing: discovery of additional chemical and biological functions for 3-oxo-n-acylhomoserine lactones. *Proceedings of the National Academy of Sciences* 102, 309–314

Milo, R., Jorgensen, P., Moran, U., Weber, G., and Springer, M. (2010). Bionumbers—the database of key numbers in molecular and cell biology. *Nucleic acids research* 38, D750–D753

Schomburg, I., Jeske, L., Ulbrich, M., Placzek, S., Chang, A., and Schomburg, D. (2017). The BRENDA enzyme information system–From a database to an expert system. *Journal of Biotechnology* 261, 194–206. doi:10.1016/j.jbiotec.2017.04.020

Siedler, S., Stahlhut, S. G., Malla, S., Maury, J., and Neves, A. R. (2014). Novel biosensors based on flavonoid-responsive transcriptional regulators introduced into escherichia coli. *Metabolic engineering* 21, 2–8
